# Stage-based colorectal cancer prediction on uncertain dataset using rough computing and LSTM models

**DOI:** 10.1038/s41598-024-77302-z

**Published:** 2024-11-21

**Authors:** K. Supriya, A. Anitha

**Affiliations:** School of Computer Science Engineering and Information Systems, Vellore Institute of Technology, Vellore, 632014 India

**Keywords:** Colorectal cancer prediction, Rough set on fuzzy approximation space (RSFAS), LSTM, Survival analysis, Weibull distribution, Computational models, Data mining, Data processing, Machine learning

## Abstract

Artificial intelligence (AI) is an attractive field of Computer Science that helps to classify and to predict various real-time applications. Perhaps AI has a major role in predicting diseases at an early stage based on history. As cancer is one of the most harmful diseases where the mortality rate is high, it is now essential to utilize the benefits of AI to have an early diagnosis of cancer. Among various cancers, Colorectal cancer (CRC) is a common form of gastrointestinal cancer, and its treatment is lengthy and costly, with a high recurrence rate and high fatality rate. Initial disease analysis and prognosis are required to improve the patient’s treatment with a better survival analysis. However, the disease prediction process depends on the collected data, where the data may contain uncertainty. Uncertain data leads to wrong predictions. Thus, it is essential to utilize rough computing, a mathematical tool to deal with uncertainty. This paper has made an effort, to handle uncertainty using a rough set of fuzzy approximation space as pre-processing and utilized Unidirectional and Bidirectional LSTM for the classification and prediction process. Thus, to demonstrate improved predictive accuracy, the proposed model adapted the optimizers and evaluated using benchmarking techniques in predicting stage-based survival rate. The comparative analysis shows that the proposed model performs well against the state-of-the-art models and can help the medical practitioner to detect CRC at an early stage and reduce the mortality rate among human beings.

## Introduction

Cancer is the deadliest disease. The International Agency for Research on Cancer (IARC) expected 10 million fatalities and 19,300,000 new cases of cancer in 2030. Among all Cancers, Colorectal Cancer (CRC) ranked as the fourth most predominant sort of cancer^1^. The high mortality rate of CRC often goes undetected until it has spread to other organs and develops into a chronic stage. Cancer can be diagnosed based on clinical stages, irrational characteristics, and tumor cells as per the medical records^2^. CRCs are generally classified using the following two predominant stages: Duke Stage and Tumor Node Metastasis (TNM) Stage^3^. The CRC diagnosis process includes various tests such as stool tests, colonoscopy, FIT-DNA tests, and much more. However, finding the early detection process of CRC can be done using the gene expression^4^too. The mapping of the tumor with the gene expression can be utilized with the help of microarray data that provides a clear picture of the molecular state of the patients required for the classification and prediction process. Thus, the clinical data and gene expression may be useful for predicting CRC among patients in the early stages.

Colorectal cancer is a heterogeneous process caused by a series of genetic, epigenetic, and molecular changes in the cells that form the lining of the colon. The factors contributing to these changes are dietary factors, environmental factors, metagenome of microbiotic factors, and the immunological response of the genome domain. In the transition from adenoma to carcinoma, oncogenes such as KRAS, BRAF, PIK3CA, APC, P53, and PTen^5,6^ play a crucial role. The gradual progression may be considered as an example of sequential tumor genesis. The gene mutations associated with conventional adenomas are APC (Adenomatosis Polyposis Coli), PIK3CA, and TP53 an acquired mutation that occurs in cancer cells, BRAF is associated with serrated lesions called sarcomas, and KRAS is associated with the RAS or MAPK pathways. After a genetic mutation, the formation of aggressive cancer cells takes place in due course of time. To deal with this heterogeneity, gene expression classifiers can be employed.

Advancements in the healthcare industry have facilitated the collection of huge data as medical history. However, extracting information from massive amounts of data is essential and meaningless unless it offers specific information. It is a well-known fact that using the information at the right time and right place improves the understanding of prediction systems. The recent developments of Artificial Intelligence (AI), plays a vital role in the automated prediction of diseases. One of the better-automated applications of AI includes machine learning and deep learning techniques. As colorectal cancer prediction depends on the occurrences of the tumors over the lifespan of humans, it is necessary to adopt a technique that helps to deal with the huge time-series data to provide a stage-based survival analysis process. Since the data collected from various health sources may contain uncertainty, to remove uncertainties, it is necessary to apply intelligent techniques to improve the consistency of the data. An effort has been made in this work using the Variance Inflation Factor (VIF) to extract significant features from the collected dataset. In addition, to predict the survival analysis the collected data should contain the event occurrences. Since every information system may not record the event, it can be captured using the Wei-bull distribution. To handle uncertainty, Pawlak ^7^ introduced a mathematical model to capture imprecision using a boundary approach called Rough set theory has been utilized. Rough set theory can be extended with fuzzy space as the Rough set on Fuzzy Approximation Space (RSFAS)^8–10^ is utilized for data transformation to maintain consistency. The consistent gene expression data is then applied to deep learning models such as Uni and Bidirectional LSTM for better classification and prediction process.

The contributions are highlighted as follows:

- Significant attributes are identified with the help of the Variance Inflation Factor in clinical and gene-based colorectal cancer dataset.
- Data consistency is maintained by handling uncertainty on employing a Rough Set on Fuzzy Approximation Space
- Better prediction accuracy of colorectal cancer is obtained as the impact of tuning hyperparameter with four optimizers using Unidirectional and Bidirectional LSTM
- Ten years survival rate has been obtained using gene values by employing Wei-bull distribution

The rest of the paper is articulated as follows: The related works discussed the research directions of previous studies on feature selection and prediction processes followed by the background fundamentals. The proposed research design section describes the feature engineering phase, data transformation phase, prediction phase and the work carried out with the empirical study on Colorectal cancer. The experimental analysis section discusses analysis done using the LSTM models followed by the comparative analysis section to provide the performance analysis of the proposed work. A discussion on stage-based survival analysis was provided as a separate discussion section followed by a conclusion and future enhancement of the proposed work.

## Related works

This section provides the related studies of various techniques and research directions applied to the Colorectal Rectal Cancer prediction process.

### Related studies on data transformation methods and feature selection processes

A feature selection is used to improve the classification performance of huge biological datasets. Some of the research directions on feature extraction are provided to predict colorectal cancer (CRC) using clinical data and identify the effective genes that contribute to CRC progression. Furthermore, deep learning can forecast disease risk for specific treatments using clinical, disorganized, large, and complex datasets. Pan et al. (2023) proposed a filter-based relief algorithm and copula entropy for feature selection with high relevance on gene expression data^11^. Alrefai et al. (2022) proposed an ensemble filter method using Chi-square, information gain, and relief for feature selection on colon cancer microarray data^12^. Saha et al. (2021) explained statistical learning methods used for independent gene selection and proposed a filter-based robust and supervised stable gene collection algorithm along with attribute selection algorithms involving symmetric uncertainty, gain ratio, Kullback-Leibler (KL) divergence, relief, and SVM-RFE, for various cancer gene expression data in terms of solidity and classification precision^13^. Mulenga et al. (2021) applied the Cbrt (cubic root) normalization method along with Minimum-Maximum Normalization (MMN) to probiotic data for colorectal cancer. The deep neural network (DNN) was utilized to achieve superior results compared to Z-Score^14^. Bhambri et al. (2021) used Leukaemia, and colon cancer gene expression datasets and applied them to the NB, SVM, and KNN classifiers for the prediction process^15^. Kumar and Halder^16^ proposed a hybrid model using a greedy fuzzy vaguely quantified rough approach for feature (gene) selection (GFVQRFS) technique with machine learning for colorectal cancer prediction. Kumar et al.^17^ proposed a semi-supervised fuzzy rough-based extreme learning machine (SSFRELM) method to enhance classification accuracy using fuzzy rough set theory to predict cancer.

**Merits:**

- The classification algorithms are capable of handling non-linear relationships in data.
- The normalization technique enables the linear transformation of data to maintain collinearity.

**Demerits:**

- Normalization is inadequate to handle outliers.
- The Feature selection process was tedious with a huge attribute set.

### Correlated studies on hybridized deep learning methods and survival analysis

Fleming et al. (2000) discussed Survival Analysis (SA) using log-rank statistics and measured the proportional hazards of covariates using the Cox statistical technique^18^. Kundu et al. (2021) discussed ensemble models to handle both linear and non-linear data^19^. Spooner et al. (2020) discussed the SA using machine learning regression models and Cox proportional models for dementia patients to handle censored data^20^. Zhao et al. (2021) presented DeepOMIX, an adaptable and explainable multi-omics DL context achieving better performance for cancer survival analysis along with the Kaplan-Meier curve and CoxPH performance of protein expression data^21^. Tasdelen et al. (2021) utilized a hybrid CNN-LSTM model designed for pre-miRNA classification to construct RNA nucleotides and obtained better prediction^22^. Rezaee et al. (2022) utilized a stacked deep autoencoder algorithm to identify the genes that are used to recognize malignant tissue from healthy tissues^23,24^.

Benefits:

- Combining deep learning and survival analysis improves the accuracy of survival predictions.
- The hybridization of deep learning with survival analysis helps to determine an individual’s risk failure by learning non-linear relationships between features in the dataset.

Drawbacks:

- Deep learning models are time-consuming to train, making them difficult to apply in real time.
- Using deep learning models on small or noisy datasets may lead to overfitting and poor performance.

From the above studies, the following research gaps are identified:-

- Early-stage prediction of cancer using machine learning technique is not accurate.
- Computational complexity is high.
- Stage-based prediction was not carried out for certain diseases.

Thus, the primary objective of this study is

- To predict the stage-based survival analysis of colorectal cancer patients with significant attributes using Variance Inflation Factor
- To obtain potential knowledge by employing a Rough Set on Fuzzy Approximation Space (RSFAS) to get almost indiscernible classes and to maintain data consistency.
- To train and test the consistency data using Uni and Bidirectional LSTM with four optimizers to obtain better prediction accuracy.
- To identify the influential gene values at various stages of colorectal cancer with a ten-year survival rate using Wei-bull distribution

## Background fundamentals

In recent years, data handling and processing to discover knowledge has taken up a major importance in various application domains. Mining data to obtain knowledge from real-world problems cannot be dealt with the simple classification concept. Thus, it is essential to know about the information system. In addition, the data collected from the real world may be inconsistent due to the insignificant attributes. Thus, there is a need to do pre-processing. In this study Variance inflation factor ( VIF) is utilized to find the significant features, Weibull distribution (WD) is used to find the event for the uncensored data, and Rough Set on Fuzzy Approximation Space (RSFAS) is applied for the data transformation. This section deals with the background fundamentals of the techniques utilized with examples appropriately.

**Information System (IS)**: Let $\zeta$ be the Universe, *IS* be the information system represented by a quadruple $IS = \textit{(}\zeta , A, \textit{Val}, f)$. Let $\zeta = (P_{1}, P_{2}, P_{3}, \ldots , P_{n})$ be a finite set of objects and $A = (a_{1}, a_{2}, a_{3}, \ldots , a_{n}) = \varnothing$ be a finite set of attributes. For every $a\in {{A}}$; *Val* is the set of values, $\text {Val} = \bigcup _{a \in A}\text {Val}_a$, where $\text {Val}_a$ is the domain of the attribute $a\in {{A}}$. The mapping $f:\zeta ~\times ~A$ is an information function such that $f(x,a)\in \text {Val}_a \forall a\in {{A}}$ and if ${{A}=C{{A}}\bigcup B{{A}}}$. Where *C* *A* : Conditional attribute and *B* *A* : is a target attribute. A sample information system is presented in Table 1 explains the information system of patient dataset indicated as $\{{{P}_{1}},{{P}_{2}},{{P}_{3}},{{P}_{4}},{{P}_{5}}\}$ with the clinical data and gene expression profiles of Serous Ovarian Cancer. The conditional attributes in clinical data are Age, Sex represented as $(a_1, a_2)$. Where the gene expression values are ${{a}_{3}}$ represents 1552256_a_at, ${{a}_{4}}$ represents 1552283_s_at, ${{a}_{5}}$ represents 1552330_at and Survival period *d* in months represents decision attribute. To perform classification, it is necessary to find the significant attributes using the variance inflation factor.

**Table 1:** Information system.

Objects (Patients)	Sex ($a_1$)	Age ($a_2$)	1552256_a_at ($a_3$)	1552283_s_at ($a_4$)	1552330_at($a_5$)	*d*
$P_1$	45	1	3.09	6.29	4.50	46
$P_2$	35	2	4.04	8.54	3.83	54
$P_3$	65	1	5.01	6.34	2.76	27
$P_4$	36	1	4.02	8.46	4.45	45
$P_5$	64	2	3.04	9.24	3.91	28

### Variance Inflation Factor (VIF)

Identifying the correlation among the attributes provides the relationship which helps in a better prediction process. The VIF is a statistical computation used to calculate the presence of multicollinearity across predictor variables on an information system^25^. The presence of high multicollinearity can lead to conflicting estimations of coefficients, hence raising challenges in the interpretation of the model’s outcomes. The VIF calculation for the jth attribute is given in Eq. (1):

1 $$\begin{aligned} VIF = \frac{1}{{1 - R_j^2}} \end{aligned}$$

Where $R_j^2$ is the result of a regression analysis between the dependent predictor variable and all other independent predictor variables. The independent variables are denoted as $v_{j}$, $1 \le v_{j} \le n$. The threshold value of VIF is denoted as $T_h$ to select the features. VIF will be calculated for each predictor variable in the information system, resulting in VIF values. Every domain has its threshold values. For clinical and gene expression data, the VIF values are given below.

$$VIF = \left\{ \begin{array}{cl}< 10 & : Indicates\;very\;low\;multicollinearity\;and\;is\;generally\;accepted \\ 10 \le VIF < 15 & :Indicates\;moderate\;multicollinearity;it\;maybe\;manageable\; \\ & \; \; depending\;on\;the\;context \\ VIF \ge 15 & : Indicates\;high\;multicollinearity:can\;consider\;addressing\;it\; \end{array} \right.$$

### Weibull distribution applied to the reduced dataset

Every object in the information system has a period of survival. If the objects are not recorded with its survival analysis process for the considered period, lacks in identifying the event association with this, thus resulting in missing the event information. But for the time series data, finding the event is of utmost importance for the classification and prediction process. Thus, Weibull distribution is considered to find the event information for the censored data. Generally, the Weibull Probability Density Function (W-PDF) is modeled for right-censored data and can be modeled in survival analysis in the following Eq. (2):

2 $$\begin{aligned} f\left( {b,\eta ,\lambda } \right) = \left\{ {\begin{array}{*{20}{c}} {\frac{\eta }{{{\lambda ^\eta }}}{x^{\eta - 1}}*{e^{ - \left( {\frac{x}{\lambda }} \right) }}^\eta ,\;\;\;x \ge 0}\\ {\begin{array}{*{20}{c}} \;\\ {0,\;\;\;\;\;\;\;\;\;\;\;\;\;\;\;\;\;\;\;\;\;\;\;\;\;\;\;\;\;\;\;\;x < 0} \end{array}} \end{array}\;\;} \right. \end{aligned}$$

where

- *b*: The independent variable of PDF.
- $\eta > 0$ - The scaling parameter: Defines the distribution’s form.
- $$\lambda$$: Shape parameter $$\lambda > 0$$

A Weibull distribution gives a distribution in which failure rates are proportional to powers of time when *b* is a period of failure.

If:

$\lambda <1$ specifies failure rate reduces over time.

$\lambda =1$ specifies failure rate is constant over time.

$\lambda >1$ specifies failure rate increases within time.

A statistical technique such as the Maximum Likelihood Estimation (MLE) is typically retained to approximate parameters $\eta$ and $\lambda$ based on the available data while taking censored observations into account, So that right-censored data can be accommodated within the Weibull distribution method by considering the mean of all the data. The Weibull Cumulative Distribution function (W-CDF) is determined using the Eq. (3)^26^.

3 $$\begin{aligned} f\left( {x,\eta ,\lambda } \right) = 1 - {e^{ - \left( {\frac{x}{\lambda }} \right) }}^\eta , Where: \eta = \frac{mean}{EXP(GAMMALN\left( {1+\frac{1}{\lambda }}\right) } \end{aligned}$$

It is expected that the patient data of sample $P_j$ accompany the Weibull distribution with shape parameter ${{\lambda }_{j}}$ and scale parameter ${{\eta }_{j}}$. The MLE, W-PDF and W-CDF are estimated for each patient using eqs. (2 and 3) to the sample information system and the working process was explained with an example in the section feature engineering phase.

### Basics of rough set on fuzzy approximation space (RSFAS)

Knowledge discovery is the process of identifying the patterns to extract knowledge from the enormous collected data. Poor data collection leads to incorrect predictions. To deal with uncertainty and categorize data can be done by the power of the knowledge extraction process using Rough Set theory. Rough Set theory is a mathematical tool that deals with uncertainty introduced by Pawlak (1991)^7^. Rough Set works with the concept of indiscernibility relation. The basics of indiscernibility relation is typically characterized by the equivalence relation. Rough Set helps to find the approximation of a crisp set using equivalence classes. Since the values of the attributes collected with the real-time dataset are not exactly the same but almost the same. Such type of attribute values cannot be processed using the equivalence relation, the concept of fuzzy tolerance relation introduced by De (1990)^27^ was utilized on Rough set as Rough Set on Fuzzy Approximation Space (Tripathy, 2009)^28,29^.

#### Rough set on fuzzy approximation space

Consider $\zeta$ to be a Universe. A Fuzzy relation ${\Re }$ is defined as a subset of $\left( {{\zeta }}~\times ~{{\zeta }}\right)$ is said to be proximity relation if ${\mathscr {L}}\left( ~{{a}_{i}},~{{a}_{j}} \right) =1$, $\forall ~{{a}_{i}}\in {{\zeta }}$ and ${\mathscr {L}}\left( ~{{a}_{i}},~{{a}_{j}} \right) =~{{\mathscr{L}}\left( ~{{a}_{j}},~{{a}_{i}} \right) }$ for ${{a}_{i}},~{{a}_{j}}\in {{\zeta }}$. Then for a given fuzzy proximity $\alpha \in \left[ 0,1 \right]$, two elements ${{a}_{i}},{{a}_{j}}$ are $\alpha -similar$ with respect to $\Re$ if $\mathscr {L}\left( ~{{a}_{i}},~{{a}_{j}} \right) \ge \alpha$ and we write $a_{i}, a_{j}$ in $\Re _\alpha$ or ${{a}_{i}}{{\Re }}{{a}_{j}}$. The two objects ${{a}_{1}} and {{a}_{2}}~$are $\alpha -identical$ with respect to $\Re$ if either ${{a}_{1}} and {{a}_{2}}$ are $\alpha -similar$ or ${{a}_{1}} and {{a}_{2}}$ are transitively $\alpha -similar$. The $\alpha$- equivalence classes thus generated is represented as $\mathcal {\Re }^{*}_{\alpha }$. The pair $K=\left( {U}, {\Re } \left( \alpha \right) \right)$ is said to be fuzzy approximation space.

Consider a target set $X \subseteq U$. Then the $\alpha$-lower and $\alpha$-upper approximation of *X* in the knowledge base *K* is expressed as below in Eqs. (4 and 5).

4 $$\begin{aligned} X_L^\alpha= & \bigcup \{ Z \in \mathcal {\Re }_\alpha ^* \mid Z \subseteq X \} \end{aligned}$$

5 $$\begin{aligned} X_U^\alpha= & \bigcup \{ Z \in \mathcal {\Re }_\alpha ^* \mid Z \cap X \ne \emptyset \} \end{aligned}$$

From the target set *X*, if $X_{U}^{\alpha } \ne X_{L}^{\alpha }$, then we can say that *X* is $\alpha$-rough. Similarly, if $X_{U}^{\alpha } = X_{L}^{\alpha }$, we say that *X* is $\alpha$-definable. The boundary region is defined as ${BND_{\mathcal {\Re }}^{\alpha }} = {X_{U}^{\alpha }} - {X_{L}^{\alpha }}$. The target set *X* is said to be $\alpha$-rough if ${BND_{\mathcal {\Re }}^{\alpha }} \ne \phi$. Where ${\mathcal {\Re }_{\alpha }^{*}}$ denotes the almost equivalence classes obtained on imposing $\alpha$-cut. Let us assume $X \subseteq U$, and $\alpha \in [0,1]$. The set *X* is said to be $\alpha$-rough if and only if ${X_{U}^{\alpha }} \ne {X_{L}^{\alpha }}$. Similarly, if ${X_{U}^{\alpha }} = {X_{L}^{\alpha }}$, we say that *X* is $\alpha$-discernible.

The almost similarity between two objects $a_{i}$ and $a_{j}$ is defined in Eq. (6) , where ${\mathscr{L}}$ represents the membership between two objects $a_{i}$ and $a_{j}$, and ${Val_{b_i}^{a_i}}$ is the value of the object $b_i$ for the attribute $a_i$. For example, on a particular duration, the collected gene expression values for two patients such as 3.04 and 3.09 are almost identical from Table 1. The reason behind this is that the two patients’ information is characterized by nearly the same information by the knowledge of the information system. However, such type of attribute values cannot be processed using the equivalence relation. The degree of belongingness between these attribute values is computed as Eq. (7) below.

6 $$\begin{aligned} {\mathscr{L}}_\Re ^{a_i}{\left( {{x_i},{x_j}} \right) }= & 1 - \frac{{\left| {Val_{{x_i}}^{{a_i}}, Val_{{x_j}}^{{a_j}}} \right| }}{{\mathrm{{max}}\left( {Val_{x_i}^{{a_i}}, \;Val_{x_j}^{{a_j}}} \right) }} \end{aligned}$$

7 $$\begin{aligned} {\mathscr{L}}_\Re ^{a_3} {\left( {P_i},{P_j} \right) }= & 1 - \frac{{\left| 3.09 - 3.04 \right| }}{{\mathrm{{max}}\left( 3.09, 3.04\right) }} = 0.98 \end{aligned}$$

This indicated that these two attribute values are almost indiscernible and their degree of belongingness is 98%. Since we are thinking of almost similarity, the degree of belonging must be higher. On considering the degree of belongingness to be 100%, it reduces to a rough set. When the value of $\alpha$ (degree of belongingness) is decreased more and more the number of classes becomes indispensable. The membership functions have been premeditated such that their values lie between [0, 1] and additionally these functions must be symmetric. Thus the uncertainty is handled. In addition, the quantitative information system can be quantified into a qualitative information system for further analysis and integrated with LSTM models. As LSTM cells can preserve the knowledge from the past information could help to predict the future event. So the LSTM models are well suited for the stage-based survival analysis of colorectal cancer patients. Thus the consistent data is further trained and tested using Unidirectional and Bidirectional LSTM models.

## Proposed research design

**Fig. 1:**
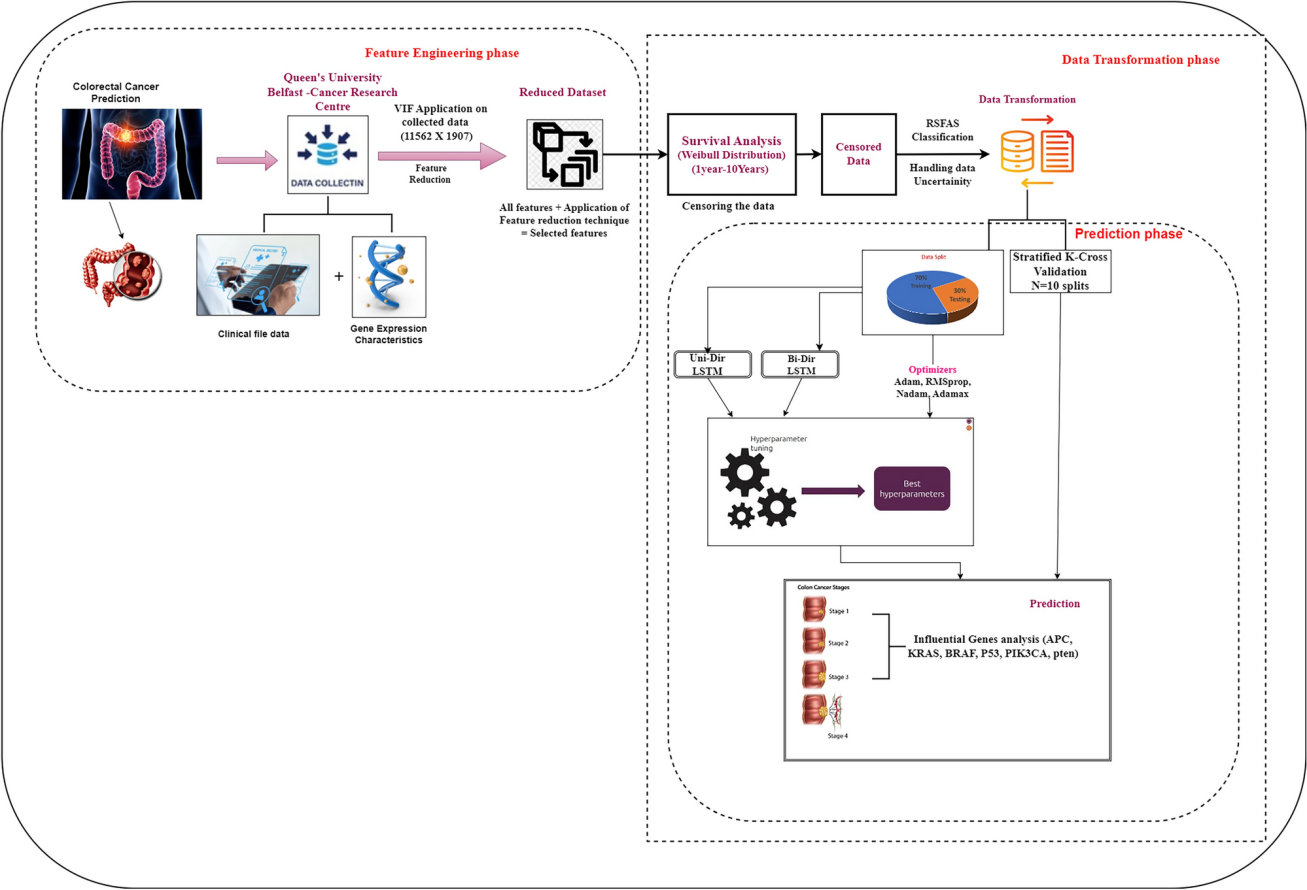
Proposed Prediction Model.

This section deals with the proposed research model development consisting of three phases: feature engineering, data transformation, and prediction. Clinicians need AI for diagnosis for several important reasons, beyond just reducing the time required for diagnosis and addressing inter-observer differences:

- Inter-observer difference: Manual diagnosis can result in significant differences in interpretation among clinicians, even when evaluating the same patient data. AI algorithms provide consistent and objective assessments, minimizing variability and enhancing diagnostic accuracy.
- Diagnostic Accuracy: AI systems can analyze complex datasets with high precision, identifying patterns and correlations that may not be immediately evident to human clinicians. This can lead to earlier and more exact diagnoses, improving patient treatment.
- Time Efficiency and Handling Large Data Volumes: AI can process large dimensions of data rapidly, significantly reducing the time required for diagnosis. This allows clinicians to focus more on patient care and other critical tasks, increasing complete efficiency in healthcare delivery.
- Early Detection and Prognosis: AI can assist in the early detection of diseases and provide predictive information, enabling timely interventions that can advance patient outcomes and potentially save lives.

The first step in developing a research model is identifying a research challenge. Furthermore, the data is subjected to knowledge discovery processes, including data collection and preparation, data cleaning, data transformation, and data partition as training and testing to find patterns that predict previously unknown associations. In the feature engineering phase, we utilized the Variance Inflation Factor (VIF) to identify the significant attributes followed by the Wei-bull distribution to identify the event for the censored data. The uncertainty was better handled using a rough set on fuzzy approximation space by transforming the qualitative data to quantitative data using fuzzy proximity relation represented as the data transformation phase. Finally, the consistent data is applied to deep learning techniques such as Unidirectional and Bidirectional LSTM to predict the survival process. A medical diagnosis related to colorectal cancer is considered for the model analysis. An abstract view of the proposed research is designed in Fig. 1.

### Feature engineering phase

In recent years, huge data have been collected through various sources. However, analyzing these data to obtain insightful information is a challenging task. In addition, identifying the attributes that play a major role in making decisions are called significant attributes. Finding these significant attributes provides computational benefits in making decisions. Most of the statistical tools help in the feature selection process. Out of which Variance Inflation Factor is used to identify the significant attributes based on multi-co-linearity. The following example 1 provides the VIF value calculation and the example 2 provides the application of Weibull Distribution for the information system provided in Table 1.

#### Example 1

VIF Value calculation for each attribute

For instance, the calculation of VIF values for the attribute ***d*** was provided in Table 2.

$$\begin{aligned} R^{2}=0.921823992 \\ VIF = \frac{1}{{1 - 0.921823992}} = 12.79164961 \end{aligned}$$

On considering the information system in Table 1, after applying the Eq. (1) shows the VIF values for the various attributes listed in Table 1. Table 2 represents the significant attributes thus providing the reduced information system. From the summary of Table 2, it has been observed that attributes $a_1$, $a_2$, $a_3$, $a_4$, and $a_5$ have a $T_h <10$. The VIF value of attribute $\textit{d} = 12.79164961$ is significantly higher than the others indicating high multicollinearity. To address the multicollinearity issues and based on domain knowledge^25^, the attribute *d* should be removed from the model. The resulting significant attributes are $a_1$, $a_2$, $a_3$, $a_4$, and $a_5$ used for further analysis.

**Table 2:** VIF Values for Attributes.

Summary	Attribute					
	$a_1$	$a_2$	$a_3$	$a_4$	$a_5$	*d*
Multiple-R	0.031092507	0.336857583	0.328842388	0.28257369	0.599448568	0.960116655
R-Square	0.000966744	0.113473031	0.108137316	0.07984789	0.359338586	0.921823992
Adjusted R-Square	−0.332044341	−0.182035958	−0.189150245	−0.22686948	0.145784781	0.895765322
Standard-Error	16.94271394	0.595492055	0.886548845	1.513169512	0.648054714	3.854016296
**VIF value**	1.127972296	1.12799722	1.121248443	1.252379	1.560887895	**12.79164961**

Significant value is in bold.

#### Example 2

**Weibull distribution**

The Wei-bull distribution (WBD) using the Weibull distribution function (WDF) Eq. (8) is applied to Table 3 after performing the VIF on attributes.

- The Weibull distribution is defined by its shape parameter $\eta$ and scale parameter $\lambda$. Let a parametric survival model with the hazard function *h*(*t*) at time *t*.
  8 $$\begin{aligned} h(t \mid X) = \eta \lambda ^{\eta } t^{\eta - 1} \exp (\beta ' X) \end{aligned}$$
  where $\beta$ is a vector of regression coefficients and *X* is a vector of covariates.

- Likelihood (*L*) Ratio: Weibull distribution likelihood ratios are used in statistical hypothesis testing to compare two different models or hypotheses about the distribution’s parameters. Calculate the likelihoods for both hypotheses, create a ratio, and use the test statistic to prove it. For a given set of parameters, the *L* ratio is calculated using the Eq. (9). Where $\delta$ = event (1 is event occurred, 0 is if censored), parameters of $\theta$ are $(\eta , \lambda , \beta )$, *u* represents the variable of integration within the integral, *T* is new observed time, X: covariates that influence hazard function $(T \mid X)$ and cumulative hazard function $H(T \mid X)$.
  9 $$\begin{aligned} H(T \mid X) = \int _{0}^{t} h(u \mid X) \, du = \lambda t^{\eta } \exp (\beta ' X) \end{aligned}$$

On applying the Wei-bull distribution on the reduced sample information system it was observed that the Wei-bull could achieve a higher survival probability for each attribute and the event column can arrive as shown in Table 3.

**Table 3:** Reduced information system with event identification.

Objects (Patients)	$a_1$	$a_2$	$a_3$	$a_4$	$a_5$	event
$P_1$	45	1	3.09	6.29	4.50	1
$P_2$	35	2	4.04	8.54	3.83	1
$P_3$	65	1	5.01	6.34	2.76	0
$P_4$	36	1	4.02	8.46	4.45	0
$P_5$	64	2	3.04	9.24	3.91	0

It is possible to describe prediction effects in terms of hazard ratios, Likelihood ratios, and terms of the relative increment during the survival analysis. This reduced dataset now checks for its consistency by employing the rough set on fuzzy approximation space.

### Data transformation phase

The concept of rough set was based on the indiscernibility relation, employed on the reduced information system. This phase entails the data classification using fuzzy proximity relation. Every attribute in the dataset undergoes fuzzy tolerance relation and generates data classification producing $\alpha$-equivalences classes, where $\alpha$ represents the degree of belongingness. The $\alpha$-equivalences classes thus obtained from the Table 3 undergo fuzzy tolerance relation with the degree of belongingness as $98\%$ i.e, $\alpha$ $\ge 0.98$. The fuzzy proximity relation for the attribute $a_1$ is presented in Table 4. From Table 4 it is observed that ${\mathscr{L}}_\Re ^{a^1} {\left( {P_1},{P_1} \right) } = 1.00$; ${\mathscr{L}}_\Re ^{a^1} {\left( {P_2},{P_2} \right) } = 1.00$; ${\mathscr{L}}_\Re ^{a^1} {\left( {P_2},{P_4} \right) } = 0.98$; ${\mathscr{L}}_\Re ^{a^1} {\left( {P_3},{P_3} \right) } = 1.00$; ${\mathscr{L}}_\Re ^{a^1} {\left( {P_4},{P_4} \right) } = 1.00$; ${\mathscr{L}}_\Re ^{a^1} {\left( {P_3},{P_5} \right) } = 0.98$; ${\mathscr{L}}_\Re ^{a^1} {\left( {P_5},{P_5} \right) } = 1.00$.

**Table 4:** Fuzzy proximity relation for attribute $a_1$.

${\mathscr{L}}_\Re ^{a^1}$	$P_1$	$P_2$	$P_3$	$P_4$	$P_5$
$P_1$	1.00	0.78	0.56	0.80	0.58
$P_2$	0.78	1.00	0.33	0.98	0.36
$P_3$	0.56	0.33	1.00	0.36	0.98
$P_4$	0.80	0.98	0.36	1.00	0.38
$P_5$	0.58	0.36	0.98	0.38	1.00

Thus the objects $P_2$ and $P_3$ are $\alpha$-identical. Similarly, $P_4$ and $P_5$ are $\alpha$-identical, whereas $P_1$ is $\alpha$-identical to itself. As the attribute $a_2$ represents the gender which takes the value as 1 and 2 for male and female respectively. Thus, the fuzzy proximity relation was not computed. The fuzzy proximity relation is computed for the other attributes such as $a_3$ to $a_5$ and the computation was presented in Tables 5, 6 and 7.

**Table 5:** Fuzzy proximity relation for attribute $a_3$.

${\mathscr{L}}_\Re ^{a^3}$	$P_1$	$P_2$	$P_3$	$P_4$	$P_5$
$P_1$	1.00	0.69	0.38	0.70	0.98
$P_1$	0.69	1.00	0.69	0.99	0.68
$P_1$	0.38	0.69	1.00	0.68	0.36
$P_1$	0.70	0.99	0.68	1.00	0.68
$P_1$	0.98	0.68	0.36	0.68	1.00

**Table 6:** Fuzzy proximity relation for attribute $a_4$.

${\mathscr{L}}_\Re ^{a^4}$	$P_1$	$P_2$	$P_3$	$P_4$	$P_5$
$P_1$	1.00	0.64	0.99	0.66	0.53
$P_2$	0.64	1.00	0.65	0.99	0.89
$P_3$	0.99	0.65	1.00	0.66	0.54
$P_4$	0.66	0.99	0.66	1.00	0.88
$P_5$	0.53	0.89	0.54	0.88	1.00

**Table 7:** Fuzzy proximity relation for attribute $a_5$.

${\mathscr{L}}_\Re ^{a^5}$	$P_1$	$P_2$	$P_3$	$P_4$	$P_5$
$P_1$	1.00	0.85	0.61	0.99	0.87
$P_1$	0.85	1.00	0.76	0.86	0.98
$P_1$	0.61	0.76	1.00	0.62	0.74
$P_1$	0.99	0.86	0.62	1.00	0.88
$P_5$	0.87	0.98	0.74	0.88	1.00

From the above tables, the $\alpha$-equivalence class obtained are listed below from Eq. (10–13):

10 $$\begin{aligned} \frac{{{\zeta }}}{{\Re _{{\alpha }}^{{a_1}}}}= & \left\{ \left\{ {{P_1}} \right\} ,\left\{ {{P_2},{P_4}} \right\} ,\left\{ {P_3},{P_5} \right\} \right\} \end{aligned}$$

11 $$\begin{aligned} \frac{{{\zeta }}}{{\Re _{{\alpha }}^{{a_3}}}}= & \left\{ \left\{ {{P_1}}, {{P_5}} \right\} ,\left\{ {{P_2},{P_4}} \right\} ,\left\{ {P_3} \right\} \right\} \end{aligned}$$

12 $$\begin{aligned} \frac{{{\zeta }}}{{\Re _{{\alpha }}^{{a_4}}}}= & \left\{ \left\{ {{P_1}}, {{P_3}} \right\} ,\left\{ {{P_2},{P_4}} \right\} ,\left\{ {P_5} \right\} \right\} \end{aligned}$$

13 $$\begin{aligned} \frac{{{\zeta }}}{{\Re _{{\alpha }}^{{a_5}}}}= & \left\{ \left\{ {{P_1}},{{P_4}} \right\} ,\left\{ {{P_2},{P_5}} \right\} ,\left\{ {P_3} \right\} \right\} \end{aligned}$$

From the above equations, it is observed that the attributes are classified into a maximum of three equivalence classes, which can be categorized as Low (1), Medium (2), and High (3) with the weights for quantifying the values. Thus, the reduced information system is now available for further prediction in the proposed model. Thus, the qualitative information system in Table 8 has been reduced to the quantitative information system as represented in Table 9.

**Table 8:** Qualitative information system.

Objects(Patients)	$a_1$	$a_2$	$a_3$	$a_4$	$a_5$
$P_1$	Medium	Low	Low	Low	High
$P_2$	Low	High	Medium	Medium	Medium
$P_3$	High	Low	High	Low	Low
$P_4$	Low	Low	Medium	Medium	High
$P_5$	High	High	Low	High	Medium

**Table 9:** Qualtitative to quantitative dataset.

Objects(Patients)	$a_1$	$a_2$	$a_3$	$a_4$	$a_5$
$P_1$	2	1	1	1	3
$P_2$	1	2	2	2	2
$P_3$	3	1	3	1	1
$P_4$	1	1	2	2	3
$P_5$	3	2	1	3	2

### Prediction phase

Prediction for unseen associations in time series data is a crucial task. As the time varies the data undergoes variation causing changes in the prediction accuracy. Thus, it is essential to have a predictive model to handle the time series data and to forecast the happenings. Due to the advancement of artificial intelligence, deep learning techniques help us to make better decisions from the huge data. Thus, an effort has been made in this paper to employ the two forms of long short-term memory techniques as Unidirectional and Bidirectional LSTM models^30^.

#### Unidirectional LSTM Architecture

**Fig. 2:**
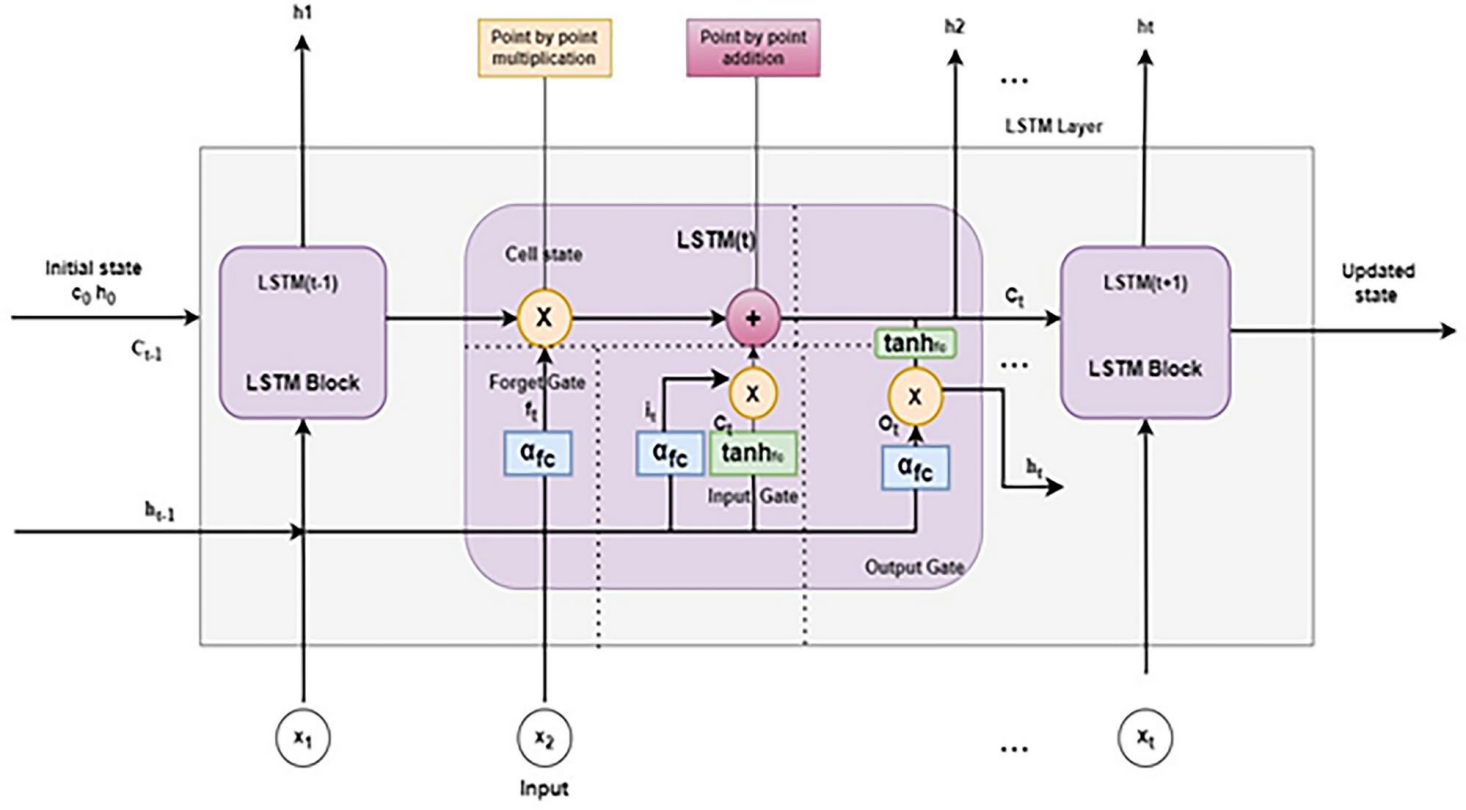
Architecture of unidirectional LSTM with multi-cell structure.

The Unidirectional LSTM units consist of feed-forward neural networks that include an input gate, forget gate and output gate. These gates help in regulating the information flow and long-term dependencies. Fig. 2 shows the Unidirectional LSTM architecture for multi-cell LSTM. LSTM is used to avoid the gradient descent problem faced by the recurrent neural network. The retention of long-term dependencies handled by $C_t$ (Memory Cell) and short-term memories handled by $h_t$ (hidden state) is based on the interaction of cells and gates, in which the flow of information is controlled. Gates use *sigmoid* activation functions to handle input and retain previous cell states, and *tanh* activation functions to store new memories. There are three gates in each cell: input gate $(i_t)$, forget gate $(f_t)$, and output gate $(O_t)$. During a time step *t*, the input gate provides new memory into the cell state, the forget gate allows the cell to forget old memory, and the output gate controls the output of the cell state and transitions to the next time step. In Fig. 2, $x_i(t)$ is an input vector at a specific time stamp *t*. $C_t$, $C_{t-1}$ are the memory cell state at specific time stamp *t* and ${t-1}$; $h_t$, $h_{t-1}$ are the hidden states at specific time step *t* and $t-1$. An input gate selects the new information to be included in the LSTM cell and the gate control is governed by its activation vector. The forget gate determines which information should be preserved in $C_{t-1}$ by utilizing the input from $x_i$ and $h_{t-1}$. The forget gate combines the hidden state value $h_t$ and input value $x_i$ using a logistic activation function sigmoid $(\sigma )$ that ranges from 0 to 1. A pointwise multiplying operation $\bigodot$ modifies the state of the preceding cell $C_{t-1}$, using the previous values. The sigmoid function $(\sigma )$ processes the hidden state value $h_{t-1}$ from the preceding layer and the input value $x_i$ from the current layer to determine the values added to $C_t$. *D* is a bias term for the input gate and the *tanh* function will be used to pass the same information about the hidden state and the current state using the activation layer at time *t*. As a result of the tanh operator, a vector is created $\widetilde{C_t}$ (updated memory cell state) with all possible values between -1 and 1. Afterward, the function converts the preceding cell state $C_{t-1}$ to $C_t$, $\widetilde{C_t}$, and $O_t$. As a result, the output values of the activation functions are ready for point-by-point multiplication. The input from the previous layer is passed on to the next layer in this structure. A layer’s final output can go to another fully connected layer for classification and prediction.

The following Eqs. (14–19) provide the gate functional process at the specific time stamp *t*. The calculation of all the above gates involves weight matrix mapping $(W_m: w_i,w_f,w_O,w_C)$ and connections $(W_m: u_i,u_f,u_O,u_C)$, as illustrated in Fig. 2.

14 $$\begin{aligned} {i_t}= & \;\left( {\sigma _{i_t}} \right) \left( {{w_i}[{x_t} + {u_i}{h_{t - 1}}] + D} \right) \end{aligned}$$

15 $$\begin{aligned} {f_t}= & \;\left( {\sigma _{f_t}} \right) \left( {{w_f}[{x_t} + {u_f}{h_{t - 1}}] + D} \right) \end{aligned}$$

16 $$\begin{aligned} {O_t}= & \;\left( {\sigma _{O_t}} \right) \left( {{w_O}[{x_t} + {u_O}{h_{t - 1}}] + D} \right) \end{aligned}$$

17 $$\begin{aligned} {\widetilde{C_t}}= & tan{h}\;\left( {{w_C}[{x_t} + {u_C}{h_{t - 1}}] + D} \right) \end{aligned}$$

18 $$\begin{aligned} {C_t}= & \;{f_t}\bigodot {C_{t - 1}} + {i_t}\bigodot {\widetilde{C_t}} \end{aligned}$$

19 $$\begin{aligned} {h_t}= & {O_t}\bigodot tan{h}\left( {{C_t}} \right) \end{aligned}$$

#### Bidirectional LSTM Architecture

A Bidirectional LSTM network is a specific type of recurrent neural network (RNN) structure designed to handle sequential data by integrating information from previous and future contexts. In contrast to Unidirectional LSTMs, even if the data processing power is high, the exclusive data process is in both directions. Bidirectional LSTMs exhibit the capability to operate in two directions and offer an advantage in addressing the issue of vanishing gradients, which is more prevalent in Unidirectional LSTM models. The backward pass of a Bidirectional LSTM involves computing gradients based on inputs and parameters to improve efficiency in capturing long-range dependencies.

**Fig. 3:**
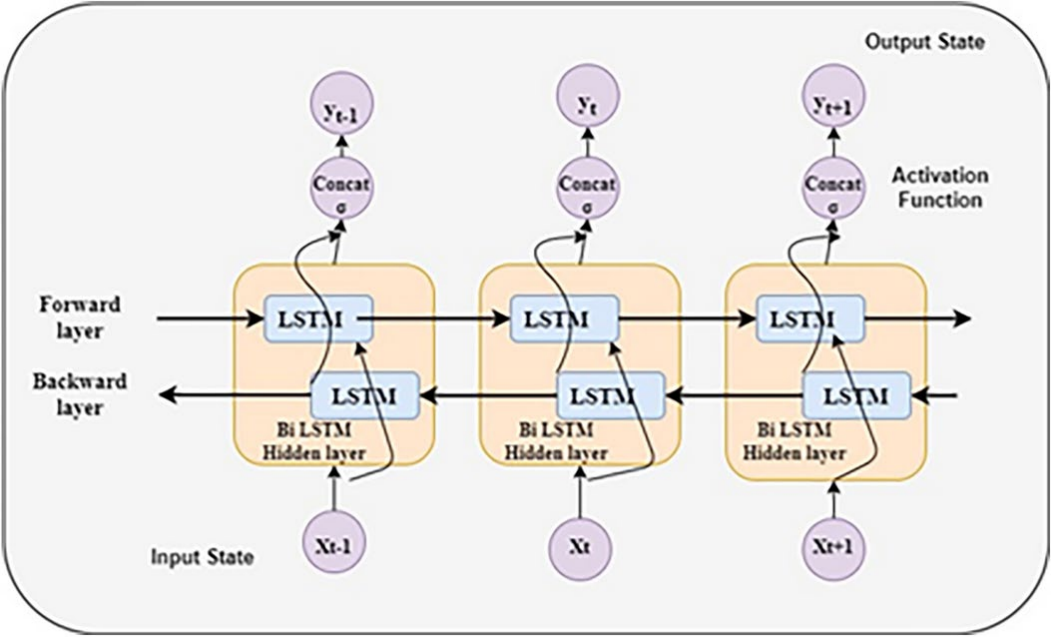
Structure of Bidirectional LSTM.

The structure of Bidirectional LSTM is represented in Fig. 3.

The total number of time steps $(T_s)$ is initialized to 1, representing the highest specific timestamp. Forward path processes input from 1 to $T_s$, while backward path processes from $T_s$ to 1. Local states at timestamps 1 and $T_s$ are set to zero. LSTM nodes process data in such a way that each input token influences the corresponding output token.

**Forward-path:** During the forward-path, the model sequentially propagates the inputs from time stamp 1 to ${{T}_{s}}$, where $1\le t\le {{T}_{s}}$, to make predictions for the output. The forward path entails bidirectional communication between time steps t = 1 and $t={{T}_{s}}$, as well as from$~t={{T}_{s}}$ to $t = 1$.

**Backward-path:** The backward path processes data over the range$~1\le t\le {{T}_{s}}$, passing through the output node. This backward path executes the sequence over the range$~{{T}_{s}}\le t\le 1$ i.e., from the end to the beginning (Table 12).

The backward path algorithm equations for gates are input gate $(i_t)$, an output gate $(O_t)$, and a candidate hidden state ${\widetilde{C_t}}$ are shown from Eqs. (20–24). At time step *t*, the total hidden state $Total_{h_t}$ is calculated by combining the current candidate hidden state and the previous hidden state:

20 $$\begin{aligned} Total_{h_t}= (1-{O_t} )\bigodot h_{(t-1)}+ {O_t} \bigodot \widetilde{C_t} \end{aligned}$$

The backward pass computation involves determining the gradients of the loss (L) function about both the network parameters and the inputs. After traversing the gates $(i_t)$ and $(O_t)$, gradients encounter the candidate hidden state ${\widetilde{C_t}}$ with partial derivative $\partial$ and the final hidden state $(h_t)$. To update the weights and biases during training, the backward path algorithm would iteratively apply these equations through time steps *t*:

21 $$\begin{aligned} \;{h_t}: \; {\partial {h_t}}{\partial {L}}= & \;{\partial _{h_t}}{\partial {L}\bigodot (1-{i_{(t+1)}})} \end{aligned}$$

22 $$\begin{aligned} \;\widetilde{C_t}: \; {\partial {G_t}}{\partial {L}}= & \;{\partial _{G_t}}{\partial {L}\bigodot {G_t}-(1-{tanh}^2\widetilde{C_t})} \end{aligned}$$

23 $$\begin{aligned} \;{i_t}: \; {\partial {i_t}}{\partial {L}}= & \;{\partial _{i_t}}{\partial {L}\bigodot (\widetilde{C_t}-h_{(t-1)})} \end{aligned}$$

24 $$\begin{aligned} \;{O_t}: \; {\partial {O_t}}{\partial {L}}= & \;{\partial _{O_t}}{\partial {L}\bigodot (1-{i_t})\bigodot {tanh}\widetilde{(C_t)}} \end{aligned}$$

## Empirical study on colorectal cancer prediction

### Data description with feature engineering process

The proposed work was applied to the collected dataset from the organization of Queen’s University Belfast from the Centre for Cancer Research and Cell Biology available open source by the National Centre for Biotechnology Information (NCBI) for colorectal cancer patients since December 2017. The dataset was characterized as clinical and gene data using geo-accession details. Every geo-accession has the 1900 gene expression values followed by age, gender, tumor location, the patient who underwent radiotherapy and chemotherapy, and disease-free survival in months comprised of six clinical attributes along with the Duke stage as the decision attribute. The Variance Inflation Factor (VIF) Eq. (1) was applied to a total of 1907 attributes to identify the significant attributes and the reduced dataset is shown in Table 11. Table 10 shows the notation representation of the collected dataset.

**Fig. 4:**
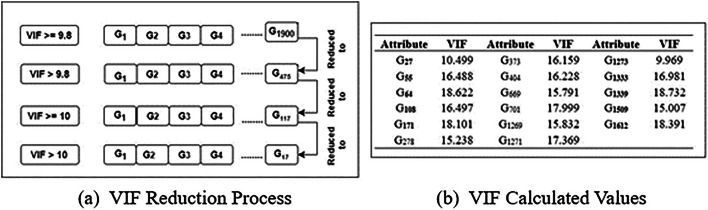
VIF Reduction process.

**Table 10:** Notation representation table.

Attribute	Abbs	Notation	PV*	MV*
Age	Age	$a_1$	[28–78]	78
Disease-free-survival (months)	DFS	$a_2$	[4–108]	108
Gender	Gender	$a_3$	[1–2]	2
Location	Location	$a_4$	[1–4]	4
Adjuvant Radiotherapy	AdjRadio	$a_5$	[0–1]	1
Adjuvant Chemotherapy	AdjChem	$a_6$	[0–1]	1
1553153_at	$G_{27}$	$a_{7}$	[3.31–5.32]	5.32
1552306_at	$G_{55}$	$a_8$	[2.76–7.26]	7.26
1552318_at	$G_{64}$	$a_9$	[3.41–6.82]	6.82
1552390_a_at	$G_{108}$	$a_{10}$	[2.79–6.01]	6.01
1552486_s_at	$G_{171}$	$a_{11}$	[5.23–8.21]	8.21
1552633_at	$G_{278}$	$a_{12}$	[4.00–5.31]	5.31
1552767_a_at	$G_{373}$	$a_{13}$	[3.32–10.35]	10.35
1552807_a_at	$G_{404}$	$a_{14}$	[5.01–8.31]	8.31
1553177_at	$G_{669}$	$a_{15}$	[2.86–5.09]	5.09
1553220_at	$G_{701}$	$a_{16}$	[3.51–6.56]	6.56
1554001_at	$G_{1269}$	$a_{17}$	[3.38–6.23]	6.23
1554004_a_at	$G_{1271}$	$a_{18}$	[3.93–6.51]	6.51
1554007_at	$G_{1273}$	$a_{19}$	[2.91–8.03]	8.03
1554095_at	$G_{1333}$	$a_{20}$	[4.03–7.61]	7.61
1554102_a_at	$G_{1339}$	$a_{21}$	[3.95–7.81]	7.81
1554341_a_at	$G_{1509}$	$a_{22}$	[3.45–5.75]	5.75
1554485_s_at	$G_{1612}$	$a_{23}$	[3.99–8.52]	8.52
Dukes stages	*d*	$a_{24}$	[0–3]	3

$^*$PV-Possible Values

$^*$MV-Maximum Values

**Table 11:** Sample dataset.

	$a_1$	$a_2$	$a_3$	$a_4$	$a_5$	$a_6$	$a_7$	$a_8$	$a_9$	$a_{10}$	$a_{11}$	$a_{12}$	$a_{13}$	$a_{14}$	$a_{15}$	$a_{16}$	$a_{17}$	$a_{18}$	$a_{19}$	$a_{20}$	$a_{21}$	$a_{22}$	$a_{23}$	$a_{24}$	$a_{25}$
Objects	Age	DFS	Gender	LOC	Adj Radio	Adj Chem	$G_{27}$	$G_{55}$	$G_{64}$	$G_{108}$	$G_{171}$	$G_{278}$	$G_{373}$	$G_{404}$	$G_{669}$	$G_{701}$	$G_{1269}$	$G_{1271}$	$G_{1273}$	$G_{1333}$	$G_{1339}$	$G_{1509}$	$G_{1612}$	*d*	event
$b_1$	75	40	2	1	2	1	4	4.55	3.65	3.31	6.47	4.5	7.05	6.6	4.24	3.99	5.79	6.26	5.79	5.9	4.63	4.64	3.99	0	0
$b_2$	61	65	2	1	2	1	4.33	3.83	4.85	3.2	6.95	4	9.24	6.49	5.1	5.13	6.23	4.65	4.46	5.3	4.05	6.86	4.91	1	0
$b_3$	62	37	1	1	2	1	3.5	2.76	3.83	4.11	6.34	5	7.98	5.48	3.02	4.8	3.9	3.93	2.91	7.17	5.65	4.03	5.3	3	0
$b_4$	47	27	1	1	2	2	4.95	4	4.02	3.48	5.59	4.6	10.35	5.81	3.49	5.21	5.52	5.53	5.04	7.2	5.04	5.1	5.14	3	1
$b_5$	70	106	2	1	1	2	3.43	3.91	4.35	2.95	5.55	4.8	8.54	6.39	3.69	3.51	4.72	5.22	8.03	6.12	4.03	5.03	5.83	2	1
$b_6$	28	85	2	2	2	2	3.95	5.73	6.82	3.71	6.16	5.2	9	7.7	2.98	3.72	4.23	4.3	5.96	7.01	6	3.76	5.88	0	0
$b_7$	54	21	2	1	2	2	3.36	4.6	4.3	2.81	6.6	5.5	7.89	8.31	5.09	4.77	4.2	6.39	4.75	5.98	4.97	4.47	4.96	2	1
$b_8$	46	64	2	2	1	1	3.85	7.26	3.72	3.8	6.13	5.3	7.65	7.49	3.61	4.12	4.5	4.53	6.93	5.3	6.2	3.65	4.16	1	0
$b_9$	61	14	2	2	2	2	3.9	4.72	3.65	5.6	7.59	4.6	9.05	7.58	3.2	4.75	4.32	4.91	5.3	5.58	4.6	5.75	6.05	1	0
$b_{10}$	55	19	2	3	1	1	4.29	3.38	3.72	4.6	5.9	4.2	8.24	5.33	3.51	4	3.83	6.26	4.93	4.21	5.53	3.67	5.26	2	1
$b_{11}$	57	36	1	1	2	1	4.01	3.43	5.36	2.95	6.29	4.7	6.73	7.21	3.81	4.53	4.97	5.01	4.62	4.03	4.39	4.15	6.92	2	1
$b_{12}$	62	56	2	2	2	1	3.3	3.68	3.4	3.96	8.21	5.2	8.44	7.19	5.01	4.4	5.69	4.75	5.36	5.7	4.46	5.07	6.82	2	1
$b_{13}$	61	63	2	2	1	2	4.01	5.08	3.95	3.38	7.19	4.9	8.05	7.09	4.23	4.16	4.83	4.86	5.92	6.25	4.67	4.78	6.23	0	0
$b_{14}$	49	17	2	3	1	1	3.65	7.26	4.1	3.21	5.51	4.7	6.1	5.97	2.45	3.94	4.16	4.26	6.93	5.7	6.02	4.1	4.91	3	1
$b_{15}$	60	38	2	4	1	2	3.8	3.53	3.99	3.3	5.53	4.4	6.73	5.57	4.24	4.88	3.7	6.61	6.88	6.3	5.5	4.24	6.75	3	1
$b_{16}$	60	16	1	1	1	2	4.2	3.75	3.47	4.6	6.06	4.9	8.35	6.12	3.36	4.3	5.31	5.95	5.13	6.01	5.33	3.96	8.52	1	0
$b_{17}$	60	38	2	2	1	2	5	4.9	4.19	3.05	7.55	4.3	9.28	6.28	2.86	5.05	5.48	4.46	4.81	5.07	7.8	6.86	6.39	0	1
$b_{18}$	73	57	2	2	2	1	3.93	3.25	4.02	4.09	6.23	4.2	6.15	5.29	3.95	4.44	4.27	5.57	5.42	5.81	4	4.49	5.01	0	0
$b_{19}$	48	108	1	3	2	1	4.21	4.15	5.44	3.41	5.57	5	8.65	6.86	3.2	4.21	4.83	5.74	5.22	5.29	5.87	4.28	5.95	1	1
$b_{20}$	71	55	2	1	2	2	3.92	5.73	3.4	5.51	6.37	5.3	6.92	6.9	3.15	5.75	4.72	4.6	4.94	7.61	7.81	5.71	5.91	2	0

According to the domain knowledge^25^, the VIF values thus obtained for various attributes and their threshold considerations with $T_h < 10$ and the attribute reduction process are shown in Fig. 4a and b, respectively. The Weibull distribution is applied to the reduced dataset and the event column is produced using the Eqs. (2 and 3). The sample dataset with the event column is represented in Table 11. The working process of the desired proposed technique is provided as Algorithm 1.

### Validating the use of Wei-bull distribution against other methods

This section discusses about the survival analysis with the statistical test performed on the collected colorectal cancer dataset. In survival analysis, model flexibility is often measured using Akaike Information Criterion (AIC) and concordance index (C-index) metrics. Weibull distributions are more flexible than exponentials because they generally have lower AICs and higher C-index scores. While log-logistic distributions are also flexible, their shape parameter can make it harder to compare the results. The Weibull distribution is clearer and can alter the magnitude of likelihood ratio metrics, offering valuable insights into hazard dynamics and enhancing predictive capabilities in survival analysis.

A statistical test was conducted using exponential, log-logistic, Cox proportional hazards, and Weibull distribution models with prognostic factors. The results in Table 12 showed that the Weibull distribution model fits better than the others, as shown by its lower AIC, a C-index of 0.85, and a higher log-likelihood ratio. Cox regression statistical tests significantly predicted colorectal cancer survival using predictor variables and deemed the Weibull proportional hazards model adequate for prediction. The Weibull distribution is superior to other normalization strategies based on previous studies, although uncertainty on collected data limits its use. Therefore, a model should reduce inconsistency on employing RSFAS.

**Algorithm 1:**
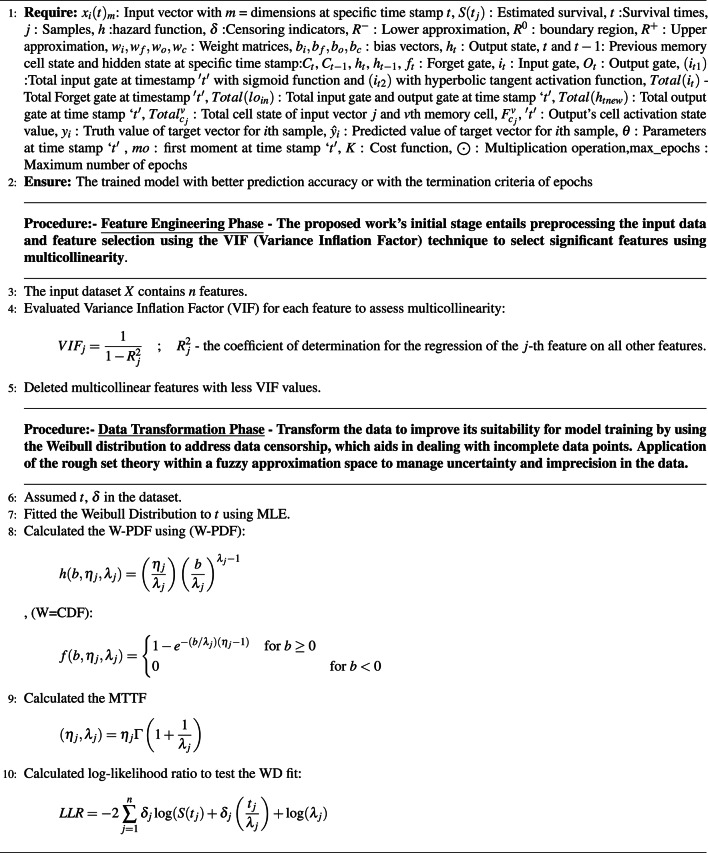
Proposed model


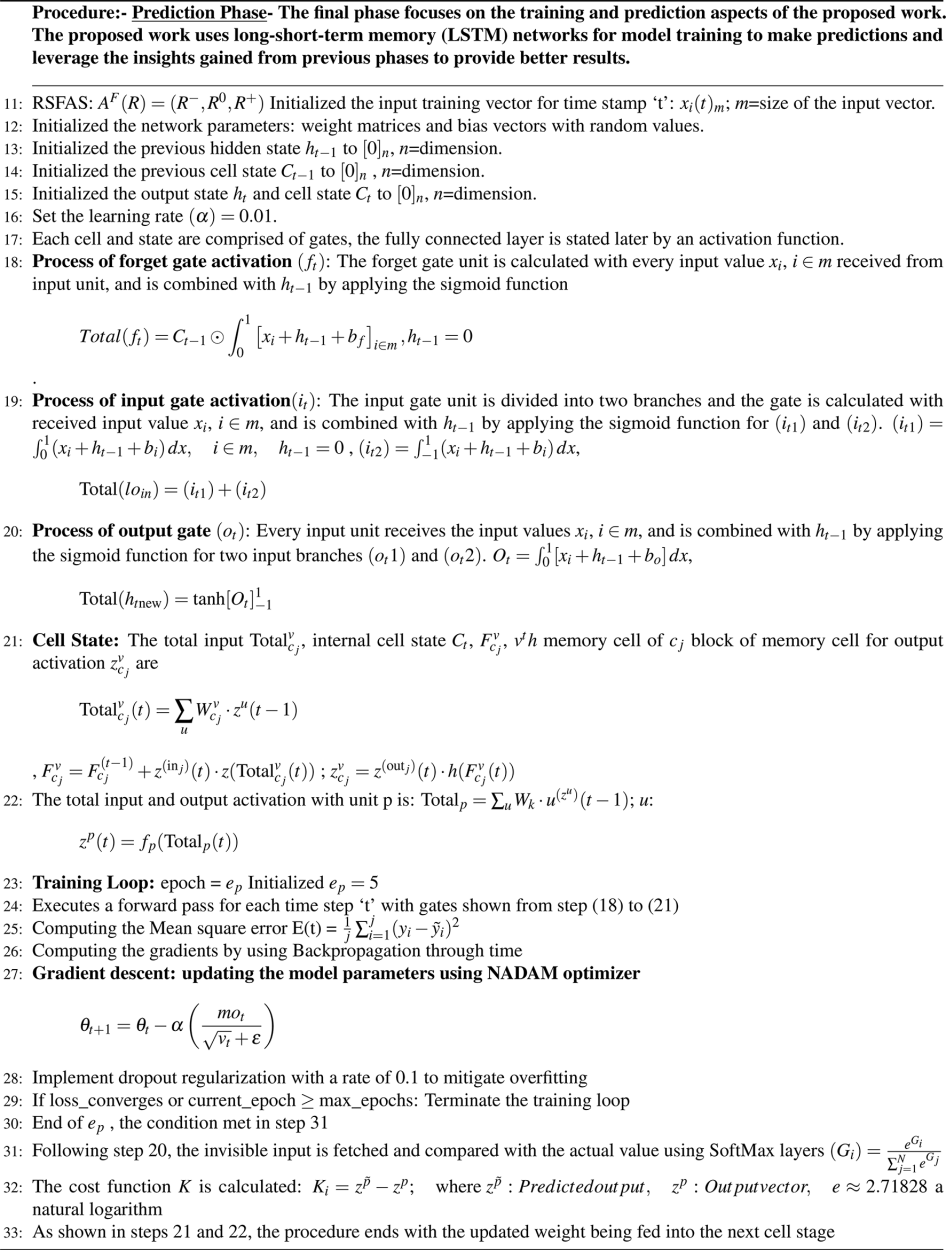


**Table 12:** Statistical analysis of VIF-Weibull model with other models.

	Exponential model	Log-Logistic model	Cox model	VIF-Weibull model
C-Index	0.69	0.60	0.76	0.85
Log-Likelihood Ratio Test	− 23436	− 36325	831.98	980.87
AIC	162463.51	158254.23	185753.83	104994.14

### Applying RSFAS to the reduced dataset

This section discusses the process of data transformation of the reduced dataset. The process of RSFAS was explained in the section Proposed Research Design. Considering that as the base, the fuzzy proximity relation was computed to the preprocessed dataset.

Let ${{\mathscr{L}}}_{\Re }^{{{a}^{i}}}$ is the fuzzy approximation relation where ${{i}}=1,2,3,4,\ldots \ldots 24$. The membership function given in Eq. (6) has been employed in the reduced dataset. The fuzzy proximity relation was computed to all the attributes, where Table 13 represents the fuzzy proximity for attribute $a_1$. On considering the almost similarity of 97% ie $\alpha \ge 97\%$, it is observed from Table 13 that ${\mathscr{L}}_{\Re }^{{{a}^{1}}}\left\{ {{b}_{1}},{{b}_{18}},{{b}_{20}},{{b}_{5}} \right\} \ge 0.97$; and ${\mathscr{L}}_{\Re }^{{{a}^{1}}}\left\{ {{b}_{2}},{{b}_{3}},{{b}_{9}},{{b}_{12}},{{b}_{13}},{{b}_{15}},{{b}_{16}},{{b}_{17}}\right\} =1.00$; ${\mathscr{L}}_{\Re }^{{{a}^{1}}}\left\{ {{b}_{4}},{{b}_{8}},{{b}_{14}},{{b}_{19}} \right\} \ge 0.97$; ${\mathscr{L}}_{\Re }^{{{a}^{1}}}\left\{ {{b}_{6}} \right\} =1.00$ and ${\mathscr{L}}_{\Re }^{{{a}^{1}}}\left\{ {{b}_{7}},{{b}_{10}},{{b}_{11}} \right\} \ge 0.97$. Hence, the objects $\left\{ {{b}_{1}},{{b}_{5}},{{b}_{18}},{{b}_{20}}\right\}$ are ${{\alpha }}-identical$. Similarly, $\left\{ {{b}_{2}},{{b}_{3}},{{b}_{9}},{{b}_{12}},{{b}_{13}},{{b}_{15}},{{b}_{16}},{{b}_{17}}\right\}$ are ${\alpha }-identical$; $\left\{ {{b}_{4}},{{b}_{8}},{{b}_{14}},{{b}_{19}}\right\}$ are ${\alpha }-identical$; $\left\{ {{b}_{6}}\right\}$ is ${\alpha }-identical$ to itself and $\left\{ {{b}_{7}},{{b}_{10}},{{b}_{11}}\right\}$ are ${\alpha }-identical.$

**Table 13:** Fuzzy Proximity relation for attribute $a_1$.

${\mathscr{L}}_R^{a^1}$	$b_1$	$b_2$	$b_3$	$b_4$	$b_5$	$b_6$	$b_7$	$b_8$	$b_9$	$b_{10}$	$b_{11}$	$b_{12}$	$b_{13}$	$b_{14}$	$b_{15}$	$b_{16}$	$b_{17}$	$b_{18}$	$b_{19}$	$b_{20}$
$b_1$	1	0.81	0.83	0.63	0.93	0.37	0.72	0.61	0.81	0.73	0.76	0.83	0.81	0.65	0.80	0.80	0.80	0.97	0.64	0.95
$b_2$	0.81	1	0.99	0.81	0.88	0.56	0.91	0.84	1	0.92	0.95	0.99	1	0.84	0.99	0.99	0.99	0.84	0.83	0.87
$b_3$	0.83	0.99	1	0.80	0.89	0.55	0.89	0.79	0.99	0.91	0.93	1	0.99	0.83	0.97	0.97	0.97	0.85	0.81	0.88
$b_4$	0.63	0.81	0.8	1	0.69	0.75	0.91	0.99	0.81	0.89	0.87	0.81	0.81	0.97	0.83	0.83	0.83	0.65	0.99	0.68
$b_5$	0.93	0.88	0.89	0.69	1	0.44	0.79	0.68	0.88	0.80	0.83	0.89	0.88	0.72	0.87	0.87	0.87	0.96	0.71	0.99
$b_6$	0.37	0.56	0.55	0.75	0.44	1	0.65	0.76	0.56	0.64	0.61	0.55	0.56	0.72	0.57	0.57	0.57	0.40	0.73	0.43
$b_7$	0.72	0.91	0.89	0.91	0.79	0.65	1	0.89	0.91	0.99	0.96	0.89	0.91	0.93	0.92	0.92	0.92	0.75	0.92	0.77
$b_8$	0.61	0.80	0.79	0.99	0.68	0.76	0.89	1	0.82	0.88	0.85	0.79	0.80	0.96	0.81	0.81	0.81	0.64	0.97	0.67
$b_9$	0.81	1	0.99	0.81	0.88	0.56	0.91	0.80	1	0.92	0.95	0.99	1	0.84	0.99	0.99	0.99	0.84	0.83	0.87
$b_{10}$	0.73	0.92	0.91	0.89	0.80	0.64	0.99	0.88	0.92	1	0.97	0.91	0.92	0.92	0.93	0.93	0.93	0.76	0.91	0.79
$b_{11}$	0.76	0.95	0.93	0.87	0.83	0.61	0.96	0.85	0.95	0.97	1	0.93	0.95	0.89	0.96	0.96	0.96	0.79	0.88	0.81
$b_{12}$	0.83	0.99	1	0.81	0.89	0.55	0.89	0.79	0.99	0.91	0.93	1	0.99	0.83	0.97	0.97	0.97	0.85	0.81	0.88
$b_{13}$	0.81	1	0.99	0.81	0.88	0.56	0.91	0.80	1	0.92	0.95	0.99	1	0.84	0.99	0.99	0.99	0.84	0.83	0.87
$b_{14}$	0.65	0.84	0.83	0.97	0.72	0.72	0.93	0.96	0.84	0.92	0.89	0.83	0.84	1	0.85	0.85	0.85	0.68	0.99	0.71
$b_{15}$	0.80	0.99	0.97	0.83	0.87	0.57	0.92	0.81	0.99	0.93	0.96	0.97	0.99	0.85	1	1	1	0.83	0.84	0.85
$b_{16}$	0.80	0.99	0.97	0.83	0.87	0.57	0.92	0.81	0.99	0.93	0.96	0.97	0.99	0.85	1	1	1	0.83	0.84	0.85
$b_{17}$	0.80	0.99	0.97	0.83	0.87	0.57	0.92	0.81	0.99	0.93	0.96	0.97	0.99	0.85	1	1	1	0.83	0.84	0.85
$b_{18}$	0.97	0.84	0.85	0.65	0.96	0.40	0.75	0.64	0.84	0.76	0.79	0.85	0.84	0.68	0.83	0.83	0.83	1	0.67	0.97
$b_{19}$	0.64	0.83	0.81	0.99	0.71	0.73	0.92	0.97	0.83	0.91	0.88	0.81	0.83	0.99	0.84	0.84	0.84	0.67	1	0.69
$b_{20}$	0.95	0.87	0.88	0.68	0.99	0.43	0.77	0.67	0.87	0.79	0.81	0.88	0.87	0.71	0.85	0.85	0.85	0.97	0.69	1

The $\alpha$-equivalence classes thus obtained for the attribute $a_1$ and $a_2$ are as shown in the following equations.

25 $$\begin{aligned} \frac{{{\zeta }}}{{\Re _{{\alpha }}^{{a_1}}}}= & \left\{ \begin{array}{l} \left\{ {{b_1},{b_{18}},{b_{20}},{b_{5}}} \right\} ,\;\left\{ {{b_2},{b_3},{b_9},{b_{12}},{b_{13}},{b_{15}},{b_{16}},{b_{17}}} \right\} ,\\ \;\;\left\{ {{b_4},{b_8},{b_{14}},{b_{19}}} \right\} ,\;\left\{ {{b_6}} \right\} ,\;\left\{ {{b_7},{b_{10}},{b_{11}}} \right\} \end{array} \right\} \end{aligned}$$

26 $$\begin{aligned} \frac{{{\zeta }}}{{\Re _{\alpha }^{{a_2}}}}= & \left\{ \begin{array}{l} \left\{ {{b_1},{b_{15}},{b_3},{b_{11}},{b_{17}}} \right\} ,\left\{ {{b_2},{b_8},{b_4},{b_{13}}} \right\} ,\left\{ {{b_5},{b_{19}},{b_{12}}, {b_{18}}, {b_{20}}} \right\} ,\left\{ {{b_6}} \right\} ,\\ \left\{ {{b_7},{b_{10}},{b_{14}},{b_9},{b_{16}}} \right\} \end{array} \right\} \end{aligned}$$

Similarly the $\alpha$-equivalence classes for the other attributes $a_7$ to $a_{23}$ are shown in the below equations from Eqs. (27–43). Since the attributes $a_3$ to $a_6$ in the dataset are already in numerical form the fuzzy proximity relation is not applied to them.

27 $$\begin{aligned} \frac{{{\zeta }}}{{\Re _{{\alpha }}^{{a_7}}}}= & \left\{ \begin{array}{l} \left\{ {b_1},{b_6},{b_8},{b_9},{b_{11}},{b_{13}},{b_{15}},{b_{18}},{b_{20}}\right\} ,\;\left\{ {{b_2},{b_{10}},{b_{16}},{b_{19}}} \right\} ,\\ \left\{ {{b_3},{b_5},{b_7},{b_{12}}} \right\} , \left\{ {{b_4},{b_{17}}} \right\} , \left\{ {{b_{14}}} \right\} \end{array} \right\} \end{aligned}$$

28 $$\begin{aligned} \frac{{{\zeta }}}{{\Re _{\alpha }^{{a_8}}}}= & \left\{ \begin{array}{l} \left\{ {{b_1},{b_7},{b_9},{b_{13}},{b_{17}}} \right\} ,\left\{ {{b_2},{b_4},{b_5},{b_{10}},{b_{11}},{b_{12}},{b_{15}},{b_{16}},{b_{18}},{b_{19}}} \right\} ,\\ \left\{ {{b_3}} \right\} ,\left\{ {{b_6},{b_{20}}} \right\} ,\left\{ {{b_8},{b_{14}}} \right\} \end{array} \right\} \end{aligned}$$

29 $$\begin{aligned} \frac{{{\zeta }}}{{\Re _{\alpha }^{{a_9}}}}= & \left\{ \begin{array}{l} \left\{ {{b_1},{b_3},{b_8},{b_9},{b_4},{b_5},{b_7},{b_{10}},{b_{13}},{b_{14}},{b_{15}},{b_{17},{b_{18}} }} \right\} ,\left\{ {{b_2}} \right\} ,\\ \left\{ {{b_6}} \right\} ,\left\{ {{b_{11}},{b_{19}}} \right\} ,\left\{ {{b_{12}},{b_{16}},{b_{20}}} \right\} \end{array} \right\} \end{aligned}$$

30 $$\begin{aligned} \frac{{{\zeta }}}{{\Re _{\alpha }^{{a_{10}}}}}= & \left\{ \begin{array}{l} \left\{ {{b_1},{b_2},{b_4},{b_5},{b_7},{b_{11}},{b_{13}},{b_{14}},{b_{15}},{b_{17}},{b_{19}}} \right\} ,\left\{ {{b_3},{b_{12}},{b_{18}}} \right\} ,\\ \left\{ {{b_6},{b_8}} \right\} ,\left\{ {{b_9},{b_{20}}} \right\} ,\left\{ {{b_{10}},{b_{16}}} \right\} \end{array} \right\} \end{aligned}$$

31 $$\begin{aligned} \frac{{{\zeta }}}{{\Re _{\alpha }^{{a_{11}}}}}= & \left\{ \begin{array}{l} \left\{ {{b_1},{b_3},{b_6},{b_7},{b_8},{b_{10}},{b_{11}}, {b_{13}} {b_{16}},{b_{18}},{b_{20}}} \right\} ,\left\{ {{b_2}} \right\} ,\\ \left\{ {{b_4},{b_5},{b_{14}},{b_{15}},{b_{19}}} \right\} ,\left\{ {{b_9},{b_{17}}} \right\} ,\left\{ {{b_{12}}} \right\} \end{array} \right\} \end{aligned}$$

32 $$\begin{aligned} \frac{{{\zeta }}}{{\Re _{\alpha }^{{a_{12}}}}}= & \left\{ \begin{array}{l} \left\{ {{b_1},{b_4},{b_9},{b_{15}},{b_{11}},{b_{14}},{b_{10}},{b_{17}},{b_{18}}} \right\} ,\left\{ {{b_2}} \right\} ,\\ \left\{ {{b_3},{b_{13}},{b_5},{b_{16}},{b_{19}}} \right\} ,\left\{ {{b_6},{b_8},{b_{12}},{b_{20}}} \right\} ,\left\{ {{b_7}} \right\} \end{array} \right\} \end{aligned}$$

33 $$\begin{aligned} \frac{{{\zeta }}}{{\Re _{\alpha }^{{a_{13}}}}}= & \left\{ \begin{array}{l} \left\{ {{b_1},{b_{11}},{b_{15}},{b_{20}}} \right\} ,\left\{ {{b_2},{b_6},{b_9},{b_{17}}} \right\} ,\left\{ {{b_3},{b_{13}},{b_{10}},{b_{12}},{b_{16}},{b_5},{b_7},{b_8}}, {b_{19}}, \right\} ,\\ \left\{ {{b_4}} \right\} ,\left\{ {{b_{14}},{b_{18}}} \right\} \end{array} \right\} \end{aligned}$$

34 $$\begin{aligned} \frac{{{\zeta }}}{{\Re _{\alpha }^{{a_{14}}}}}= & \left\{ \begin{array}{l} \left\{ {{b_1},{b_2},{b_4},{b_5},{b_{14}},{b_{16}},{b_{17}}} \right\} ,\left\{ {{b_3},{b_{10}},{b_{15}},{b_{18}}} \right\} ,\left\{ {{b_6},{b_8},{b_9}} \right\} ,\\ \left\{ {{b_7}} \right\} ,\left\{ {{b_{11}},{b_{12}},{b_{13}},{b_{19}},{b_{20}}} \right\} \end{array} \right\} \end{aligned}$$

35 $$\begin{aligned} \frac{{{\zeta }}}{{\Re _{\alpha }^{{a_{15}}}}}= & \left\{ \begin{array}{l} \left\{ {{b_1},{b_{13}},{b_{15}}} \right\} ,\left\{ {{b_2},{b_7},{b_{12}}} \right\} ,\left\{ {{b_3},{b_6},{b_9},{b_{17}},{b_{19}},{b_{20}}} \right\} ,\\ \left\{ {{b_4},{b_5},{b_8},{b_{10}},{b_{11}},{b_{16}},{b_{18}}} \right\} ,\left\{ {{b_{14}}} \right\} \end{array} \right\} \end{aligned}$$

36 $$\begin{aligned} \frac{{{\zeta }}}{{\Re _{\alpha }^{a{16}}}}= & \left\{ \begin{array}{l} \left\{ {{b_1},{b_8},{b_{10}},{b_{11}},{b_{12}},{b_{13}},{b_{14}},{b_{16}},{b_{18}},{b_{19}}} \right\} ,\left\{ {{b_2},{b_3},{b_4},{b_7},{b_9},{b_{15}},{b_{17}}} \right\} ,\\ \left\{ {{b_5}} \right\} ,\left\{ {{b_6}} \right\} ,\left\{ {{b_{20}}} \right\} \end{array} \right\} \end{aligned}$$

37 $$\begin{aligned} \frac{{{\zeta }}}{{\Re _{\alpha R}^{{a_{17}}}}}= & \left\{ \begin{array}{l} \left\{ {{b_1},{b_4},{b_{12}},{b_{16}},{b_{17}}} \right\} ,\left\{ {{b_2}} \right\} ,\left\{ {{b_3},{b_{10}},{b_{15}}} \right\} \left\{ {{b_5},{b_{11}},{b_{13}},{b_{19}},{b_{20}}} \right\} ,\\ \left\{ {{b_6},{b_7},{b_8},{b_9},{b_{14}},{b_{18}}} \right\} \end{array} \right\} \end{aligned}$$

38 $$\begin{aligned} \frac{{{\zeta }}}{{\Re _{\alpha R}^{{a_{18}}}}}= & \left\{ \begin{array}{l} \left\{ {{b_1},{b_7},{b_{10}}} \right\} ,\left\{ {{b_2},{b_5},{b_6},{b_8},{b_9},{b_{11}},{b_{12}},{b_{13}}, {b_{14}},{b_{17}},{b_{20}}} \right\} ,\\ \left\{ {{b_3}} \right\} ,\left\{ {{b_4},{b_{18}},{b_{19}},{b_{16}}} \right\} ,\left\{ {{b_{15}}} \right\} \end{array} \right\} \end{aligned}$$

39 $$\begin{aligned} \frac{{{\zeta }}}{{\Re _{\alpha R}^{{a_{19}}}}}= & \left\{ \begin{array}{l} \left\{ {{b_1},{b_6},{b_{13}},{b_{19}}} \right\} ,\left\{ {{b_2},{b_4},{b_7},{b_9},{b_{10}},{b_{11}},{b_{12}},{b_{16}},{b_{17}},{b_{19}},{b_{20}}} \right\} ,\\ \left\{ {{b_3}} \right\} ,\left\{ {{b_5}} \right\}, \left\{ {{b_8},{b_{14}},{b_{15}}} \right\} \end{array} \right\} \end{aligned}$$

40 $$\begin{aligned} \frac{{{\zeta }}}{{\Re _{\alpha }^{{a_{20}}}}}= & \left\{ \begin{array}{l} \left\{ {{b_1},{b_5},{b_7},{b_9},{b_{12}},{b_{13}},{b_{14}},{b_{15}},{b_{16}},{b_{18}}} \right\} ,\left\{ {{b_2},{b_8},{b_{17}},{b_{19}}} \right\} ,\\ \left\{ {{b_3},{b_4},{b_6}} \right\} ,\left\{ {{b_{10}},{b_{11}}} \right\} ,\left\{ {{b_{20}}} \right\} \end{array} \right\} \end{aligned}$$

41 $$\begin{aligned} \frac{{{\zeta }}}{{\Re _{\alpha }^{{a_{21}}}}}= & \left\{ \begin{array}{l} \left\{ {{b_1},{b_9},{b_{11}},{b_{12}},{b_{13}}} \right\} ,\left\{ {{b_2},{b_5},{b_{18}}} \right\} ,\left\{ {{b_3},{b_6}, {b_8},{b_{10}},{b_{14}},{b_{15}},{b_{16}},{b_{19}}} \right\} ,\\ \left\{ {{b_4},{b_7}} \right\} ,\left\{ {{b_{17}},{b_{20}}} \right\} \end{array} \right\} \end{aligned}$$

42 $$\begin{aligned} \frac{{{\zeta }}}{{\Re _{\alpha }^{{a_{22}}}}}= & \left\{ \begin{array}{l} \left\{ {{b_1},{b_3},{b_7},{b_{13}},{b_{14}},{b_{15}},{b_{16}},{b_{18}},{b_{19}}} \right\} ,\left\{ {{b_2},{b_{17}}} \right\} ,\left\{ {{b_4},{b_5},{b_{12}}} \right\} ,\\ \left\{ {{b_6},{b_8}, {b_{10}},{b_{11}}} \right\} ,\left\{ {{b_9},{b_{20}}} \right\} \end{array} \right\} \end{aligned}$$

43 $$\begin{aligned} \frac{{{\zeta }}}{{\Re _{\alpha }^{{a_{23}}}}}= & \left\{ \begin{array}{l} \left\{ {{b_1},{b_8}} \right\} ,\left\{ {{b_2},{b_3},{b_4},{b_7},{b_{10}},{b_{14}},{b_{18}}} \right\} ,\left\{ {{b_5},{b_6},{b_9},{b_{13}},{b_{17}},{b_{19}}} \right\} ,\\ \left\{ {{b_{11}},{b_{12}},{b_{15}}} \right\} ,\left\{ {{b_{16}, {b_{20}}}} \right\} \end{array} \right\} \end{aligned}$$

According to the ${\alpha }-$equivalence classes with $\ge$ 97%, the attributes can be distinguished into five classes: Very high (VH), High (H), Medium (M), Low (L), and Very low (VL) shown in Table 14 and the categories have been weighed from Very high to Very low, from 5 to 1. However, the decision attribute Dukes stages is weighted with four categories from 3 to 0 and the same is depicted in Table 15. Thus, the $\alpha$-equivalence classes provide data consistency with data quality maintained to adapt to the uncertain nature of the collected data. This consistent data is now ready to undergo experimental analysis using the LSTM techniques.

**Table 14:** Qualitative dataset.

Objects	$a_1$	$a_2$	$a_3$	$a_4$	$a_5$	$a_6$	$a_7$	$a_8$	$a_9$	$a_{10}$	$a_{11}$	$a_{12}$	$a_{13}$	$a_{14}$	$a_{15}$	$a_{16}$	$a_{17}$	$a_{18}$	$a_{19}$	$a_{20}$	$a_{21}$	$a_{22}$	$a_{23}$	*d*
$b_1$	VH	H	2	1	2	1	M	M	L	VL	L	L	L	L	H	M	H	H	M	M	L	L	VL	0
$b_2$	H	M	2	1	2	1	H	L	M	VL	L	VL	H	L	VH	H	VH	L	L	L	VL	VH	L	1
$b_3$	H	H	1	1	2	1	VL	VL	L	M	L	M	M	VL	L	H	VL	VL	VL	H	H	L	L	2
$b_4$	L	H	1	1	2	2	VH	L	H	VL	L	L	VH	L	M	H	H	M	L	H	M	M	L	3
$b_5$	VH	VL	2	1	1	2	VL	L	L	VL	VL	M	M	L	M	VL	M	L	VH	M	VL	M	M	2
$b_6$	VL	L	2	2	2	2	M	H	VH	L	L	H	H	H	VH	L	L	L	M	H	H	VL	M	0
$b_7$	M	VH	2	1	2	2	VL	M	L	VL	L	VH	M	VH	VH	M	L	H	L	M	M	L	L	2
$b_8$	L	M	2	2	1	1	M	VH	L	L	L	H	M	H	M	M	L	L	H	L	H	VL	VL	1
$b_9$	H	VH	2	2	2	2	M	M	L	VH	H	L	H	H	L	H	L	L	L	M	L	H	M	1
$b_{10}$	M	VH	2	3	1	1	H	L	L	H	L	L	M	VL	M	M	VL	H	L	VL	H	VL	L	2
$b_{11}$	M	H	1	1	2	1	H	L	H	VL	L	L	L	M	M	M	M	L	L	VL	VL	L	H	2
$b_{12}$	H	M	2	2	2	1	VL	L	VL	M	VH	H	M	M	VH	M	H	L	L	M	VL	M	H	2
$b_{13}$	H	M	2	2	1	2	M	M	L	VL	M	M	M	M	H	H	M	L	M	M	L	L	M	0
$b_{14}$	L	VH	2	3	1	1	L	VH	L	VL	VL	L	VL	L	VL	M	L	L	H	M	H	L	L	3
$b_{15}$	H	H	2	4	1	2	M	L	L	VL	VL	L	L	VL	H	H	VL	VH	H	M	H	L	H	3
$b_{16}$	H	VH	1	1	1	2	H	L	VL	H	L	M	M	L	M	M	H	M	L	M	H	L	VH	1
$b_{17}$	H	VH	2	2	1	2	VH	M	L	VL	H	L	H	L	L	H	H	L	L	M	VH	VH	M	0
$b_{18}$	VH	M	2	2	2	1	M	L	L	M	L	L	VL	VL	M	M	L	M	L	M	VL	L	L	0
$b_{19}$	L	VL	1	3	2	1	H	L	H	VL	VL	M	M	M	L	M	M	M	L	L	H	L	M	1
$b_{20}$	VH	M	2	1	2	2	M	H	VL	VH	L	H	L	M	L	VH	M	L	L	VH	VH	H	M	2

**Table 15:** Quantitative dataset.

Objects	$a_1$	$a_2$	$a_3$	$a_4$	$a_5$	$a_6$	$a_7$	$a_8$	$a_9$	$a_{10}$	$a_{11}$	$a_{12}$	$a_{13}$	$a_{14}$	$a_{15}$	$a_{16}$	$a_{17}$	$a_{18}$	$a_{19}$	$a_{20}$	$a_{21}$	$a_{22}$	$a_{23}$	*d*
$b_1$	5	4	2	1	2	1	3	3	2	1	2	2	2	2	4	3	4	4	3	3	2	2	1	0
$b_2$	4	3	2	1	2	1	4	2	3	1	2	1	4	2	5	4	5	2	2	2	1	5	2	1
$b_3$	4	4	1	1	2	1	1	1	2	3	2	3	3	1	2	4	1	1	1	4	4	2	2	2
$b_4$	2	4	1	1	2	2	5	2	4	1	2	2	5	2	3	4	4	3	2	4	3	3	2	3
$b_5$	5	1	2	1	1	2	1	2	2	1	1	3	3	2	3	1	3	2	5	3	1	3	3	2
$b_6$	1	2	2	2	2	2	3	4	5	2	2	4	4	4	5	2	2	2	3	4	4	1	3	0
$b_7$	3	5	2	1	2	2	1	3	2	1	2	5	3	5	5	3	2	4	2	3	3	2	2	2
$b_8$	2	3	2	2	1	1	3	5	2	2	2	4	3	4	3	3	2	2	4	2	4	1	1	1
$b_9$	4	5	2	2	2	2	3	3	2	5	4	2	4	4	2	4	2	2	2	3	2	4	3	1
$b_{10}$	3	5	2	3	1	1	4	2	2	4	2	2	3	1	3	3	1	4	2	1	4	1	2	2
$b_{11}$	3	4	1	1	2	1	4	2	4	1	2	2	2	3	3	3	3	2	2	1	1	2	4	2
$b_{12}$	4	3	2	2	2	1	1	2	1	3	5	4	3	3	5	3	4	2	2	3	1	3	4	2
$b_{13}$	4	3	2	2	1	2	3	3	2	1	3	3	3	3	4	4	3	2	3	3	2	2	3	0
$b_{14}$	2	5	2	3	1	1	2	5	2	1	1	2	1	2	1	3	2	2	4	3	4	2	2	3
$b_{15}$	4	4	2	4	1	2	3	2	2	1	1	2	2	1	4	4	1	5	4	3	4	2	4	3
$b_{16}$	4	5	1	1	1	2	4	2	1	4	2	3	3	2	3	3	4	3	2	3	4	2	5	1
$b_{17}$	4	5	2	2	1	2	5	3	2	1	4	2	4	2	2	4	4	2	2	3	5	5	3	0
$b_{18}$	5	3	2	2	2	1	3	2	2	3	2	2	1	1	3	3	2	3	2	3	1	2	2	0
$b_{19}$	2	1	1	3	2	1	4	2	4	1	1	3	3	3	2	3	3	3	2	2	4	2	3	1
$b_{20}$	5	3	2	1	2	2	3	4	1	5	2	4	2	3	2	5	3	2	2	5	5	4	3	2

### Experimental analysis of the proposed prediction phase

The experimental analysis was conducted using Google Colab with a 12th Intel i5 processor and 8GB RAM, on Windows 11 operating system. The collected and consistent data contains 11562 objects that undergo data partition for training and testing respectively for Unidirectional and Bidirectional LSTM. The search space and the selected choice for both models are listed in Table 16. The implementation was carried out by varying the number of units, number of batches, and with various data splits. On varying these hyperparameters, it was found that the learning rate of 0.018 suits better with 100 LSTM units for 70%-30% of the data partition for Unidirectional LSTM, whereas the learning rate of 0.015 with 90 LSTM units, of the 70%-30% of data partition provides better accuracy values for Bidirectional LSTM as it provides both forward and backward pass.

LSTM models have the ability to handle large amounts of training data to achieve good performance by remembering the information over time. At the same time suffers from vanishing gradient descent problems, which causes a low convergence rate. This can be handled by using optimizers. The proposed model checked its performance using various optimizers such as Adam, RMSprop, NADAM, and Adamax. Adam optimizer was chosen to minimize the loss function as it tends to converge faster. As both Adam and RMSProp correlate them using momentum, an improved type of momentum called Nesterov momentum^31^is added as an extension to the Adam optimizer. Similarly, the variant of the Adam optimizer was Adamax, which has the capability of adjusting the learning rate concerning the characteristics of data.

**Table 16:** The tuning hyperparameters.

Hyperparameters	RSFAS-Unidirectional LSTM		RSFAS-Bidirectional LSTM	
	Search space	Selected choice	Search space	Selected choice
Data split	(60–40)%,(70–30)%,(80–20)%	(70–30)%	(60–40)%,(70–30)%,(80–20)%	(70–30)%
Number of units	30,60,100	100	30,60,90,100	100
Number of hidden layers	1,2	1	1,2,3	2
Number of epochs	50,100,150,200	100	50,100,150,200	100
Batch size	10,32,64	64	10,32,64	64
Learning rate	0.01,0.02	0.01	0.01,0.02	0.02
Drop out	0.1,0.2	0.1	0.1,0.2	0.2
Optimizers	Adam, RMSprop, NADAM, Adamax	NADAM	Adam, RMSprop, NADAM, Adamax	NADAM

As the pre-processing was carried out using RSFAS, the consistent data was fed into the RSFAS-Unidirectional and RSFAS-Bidirectional LSTM for training and testing. From Table17, it is clearly seen that during the 100th epoch, even though RMSProp attains 94.28 accuracy, the F-score and Sensitivity are overall better for the NADAM optimizer than the RMSProp optimizer. Thus the LSTM inability for the local and global search was noted and to overcome we utilized four optimizers such as RSFAS-Adam *(Opt 1)*, RSFAS-RMSProp *(Opt 2)*, RSFAS-NADAM *(Opt 3)* and RSFAS-Adamax*(Opt 4)* are applied and the classification accuracies were noted for RSFAS-Unidirectional and RSFAS-Bidirectional LSTM for various batch sizes.

**Table 17:** Classification accuracy and value-loss—batch size—32 for uni and bidirectional LSTM.

Epochs	(RSFAS-Unidirectional LSTM) Batch size-32								(RSFAS-Bidirectional LSTM) Batch size-32							
	RSFAS-Unidirectional accuracy								RSFAS-Bidirectional accuracy							
	Adam		RMSprop		Nadam		Adamax		Adam		Rmsprop		Nadam		Adamax	
	ACC	Loss	ACC	Loss	ACC	Loss	ACC	Loss	ACC	Loss	ACC	Loss	ACC	Loss	ACC	Loss
5	89.05	0.0125	90.59	0.0418	90.72	0.0184	84.46	0.0242	90.05	0.0125	90.72	0.0418	93.24	0.0184	82.14	0.0262
10	89.42	0.0183	90.64	0.03	90.87	0.0126	84.75	0.0417	91.22	0.0183	91.37	0.03	93.36	0.0126	84.65	0.0427
20	89.76	0.0241	90.75	0.03	92.07	0.0068	86.32	0.0184	91.76	0.0241	92.37	0.03	93.41	0.0068	85.26	0.0124
30	89.82	0.0183	90.92	0.0241	92.46	0.0241	87.57	0.0068	91.82	0.0183	92.55	0.0241	93.68	0.0241	85.47	0.0068
40	90.02	0.0183	91.64	0.0068	92.67	0.0126	88.66	0.0301	92.02	0.0183	92.67	0.0068	93.89	0.0126	86.98	0.0301
50	90.26	0.029	91.74	0.0795	92.89	0.0241	88.87	0.0125	92.37	0.029	92.79	0.0795	93.52	0.0241	87.22	0.0125
60	90.57	0.029	91.88	0.0795	92.91	0.0184	89.22	0.0359	92.87	0.029	93.09	0.0795	93.83	0.0184	88.89	0.0349
70	90.47	0.0241	92.59	0.0242	92.97	0.0299	89.64	0.030	93.47	0.0241	93.66	0.0242	94.37	0.0299	89.17	0.0329
80	90.72	0.0183	92.63	0.0125	93.02	0.030	89.75	0.0184	93.62	0.0183	94.75	0.0125	94.78	0.030	89.56	0.0174
90	90.98	0.0241	92.81	0.0357	93.04	0.0068	89.94	0.0532	93.98	0.0241	94.95	0.0267	94.96	0.0068	91.22	0.0632
100	91.29	0.0357	92.87	0.0358	**93.07**	0.0261	90.22	0.0341	94.05	0.0354	95.03	0.0286	**95.43**	0.0258	91.24	0.0354

Significant values are in bold.

The analysis was carried out using various evaluation metrics such as Precision, Sensitivity (Recall), F1-score, and Specificity with the metrics such as Kappa Score, KL Divergence, and ROC-AUC Score, and its calculation formula is provided from Eqs. (44–48). Evaluation metrics, such as True Positives (TP), False Positives (FP), False Negatives (FN), and True Negatives (TN), are calculated based on the classification of the Dukes stages.

True Positives (TP): Refer to the cases where the model accurately classified the cancer stage.

False Positives (FP): The number of instances where the model inaccurately classified a distinct stage as the actual stage.

False Negatives (FN): Refer to the instances in which the model incorrectly classified a different stage as the true stage (Table 18 ).

**Table 18:** Classification accuracy and value-loss—batch size - 64 for uni and bidirectional LSTM.

Epochs	(RSFAS-Unidirectional LSTM) Batch size-64								(RSFAS-Bidirectional LSTM) Batch size-64							
	RSFAS-Unidirectional accuracy								RSFAS-Bidirectional accuracy							
	Adam		RMSprop		Nadam		Adamax		Adam		Rmsprop		Nadam		Adamax	
	ACC	Loss	ACC	Loss	ACC	Loss	ACC	Loss	ACC	Loss	ACC	Loss	ACC	Loss	ACC	Loss
5	89.21	0.034	84.97	0.0418	90.62	0.0184	84.46	0.0242	90.12	0.0237	90.84	0.0295	93.14	0.0069	82.04	0.0767
10	90.16	0.0309	86.23	0.03	90.64	0.0126	85.75	0.0417	92.64	0.031	91.82	0.0271	93.56	0.0139	84.85	0.0724
20	90.79	0.0281	87.52	0.03	90.75	0.0068	86.32	0.0184	93.71	0.0268	92.77	0.0246	93.71	0.0095	85.38	0.0678
30	90.81	0.0277	88.63	0.0241	90.96	0.0241	87.67	0.0068	93.89	0.0234	93.37	0.0213	93.99	0.0087	85.67	0.0626
40	91.43	0.0412	89.43	0.0068	91.64	0.0126	88.26	0.0301	94.07	0.0264	93.59	0.0189	94.33	0.0073	85.98	0.0576
50	91.64	0.0181	90.67	0.0795	91.77	0.0241	88.57	0.0125	94.23	0.0186	93.89	0.0182	94.49	0.0107	86.22	0.0531
60	91.83	0.0173	91.34	0.0795	91.98	0.0184	89.22	0.0359	94.55	0.0196	94.01	0.0174	94.93	0.0129	87.99	0.0474
70	92.39	0.0241	92.59	0.0242	92.69	0.0299	89.64	0.03	94.72	0.0184	94.25	0.0159	95.37	0.0111	88.17	0.0462
80	92.45	0.0183	92.67	0.0125	92.73	0.03	90.25	0.0184	95.06	0.02	94.76	0.0151	95.78	0.0093	89.75	0.0415
90	92.57	0.0241	92.86	0.0357	92.81	0.0068	91.54	0.0532	95.53	0.0162	95.39	0.009	95.96	0.0036	91.26	0.0361
100	92.61	0.0331	93.01	0.0261	**93.27**	**0.0258**	92.02	0.0271	95.67	0.0177	95.87	0.0143	**96.12**	**0.0022**	91.44	0.0342

Significant values are in bold.

True Negatives (TN): The number of cases in which the model accurately recognized that a sample was not relevant to a specific stage.

44 $$\begin{aligned} Precision= & \frac{TP}{TP+FP} \end{aligned}$$

45 $$\begin{aligned} Sensitivity= & \frac{TP}{TP+FN} \end{aligned}$$

46 $$\begin{aligned} Specificity= & \frac{TN}{TN+FP} \end{aligned}$$

47 $$\begin{aligned} F-score= & \frac{2(Precision*Recall)}{(Precision + Recall)} \end{aligned}$$

48 $$\begin{aligned} Kappa-Score= & \frac{{P_o - P_e}}{{1 - P_e}} \end{aligned}$$

where $P_0$ = Observed proportion of the agreement between rating, $P_e$ = Expected proportion of agreement between rating, and the value of the kappa score ranges from -1 ( worst than perfect chance) and 1 (perfect agreement).

49 $$\begin{aligned} KL-Divergence = D_{KL}(P \parallel Q) = \int _{-\infty }^{\infty } p(x) \log \left( \frac{q(x)}{p(x)} \right) \, dx \end{aligned}$$

$D_{KL}(P \parallel Q)$: The distribution from *Q* to *P*, where *P*(*x*) and *Q*(*x*): Probability density functions.

**Fig. 5:**
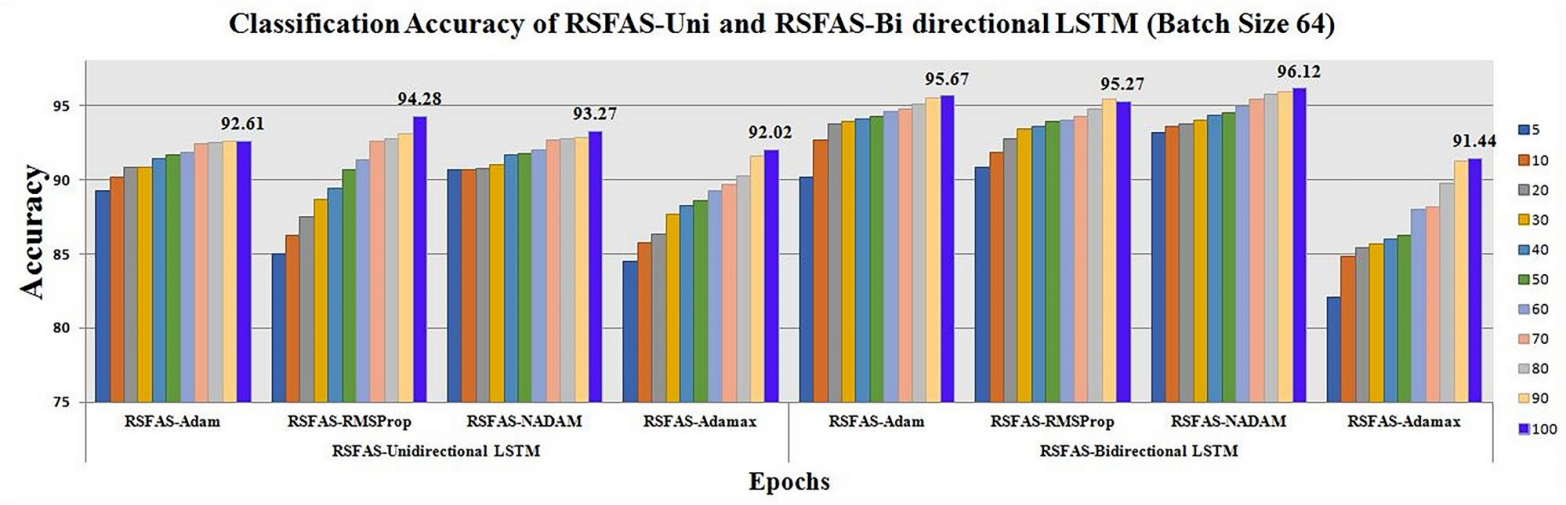
Classification Accuracy of RSFAS- Unidirectional and RSFAS-Bidirectional LSTM(Batch size-64).

A stratified K-fold cross-validation with n=10 splits was carried out with metrics such as Kappa score, KL Divergence, and AUC under receiver operating characteristic curves (ROC-AUC). The results are shown in Tables 19 and 20. Fig. 5 represents the classification accuracies of RSFAS-Unidirectional and RSFAS-Bidirectional LSTM tuned with suitable hyperparameters. ROC curves are used to estimate the number of true and false positives in the training data using confusion metrics.

**Table 19:** RSFAS-Bidirectional LSTM stratified K-fold cross validation results.

RSFAS-Bidirectional LSTM Stratified K-fold Cross Validation					
Folds	Fold-1	Fold-2	Fold-3	Fold-4	Fold-5
Accuracy (%)	99.57	99.05	99.83	99.83	100
Folds	Fold-6	Fold-7	Fold-8	Fold-9	Fold-10
Accuracy (%)	99.39	100	99.31	99.05	100

**Table 20:** Classification accuracy with various optimizers for batch size-64.

Epochs	Classification accuracy with Batch size-64							
	RSFAS-Unidirectional accuracy				RSFAS-Bidirectional accuracy			
	*Opt 1*	*Opt 2*	*Opt 3*	*Opt 4*	*Opt 1*	*Opt 2*	*Opt 3*	*Opt 4*
5	89.21	84.97	90.62	84.46	90.12	90.84	93.14	82.04
10	90.16	86.23	90.64	85.75	92.64	91.82	93.56	84.85
20	90.79	87.52	90.75	86.32	93.71	92.77	93.71	85.38
30	90.81	88.63	90.96	87.67	93.89	93.37	93.99	85.67
40	91.43	89.43	91.64	88.26	94.07	93.59	94.33	85.98
50	91.64	90.67	91.77	88.57	94.23	93.89	94.49	86.22
60	91.83	91.34	91.98	89.22	94.55	94.01	94.93	87.99
70	92.39	92.59	92.69	89.64	94.72	94.25	95.37	88.17
80	92.45	92.77	92.73	90.25	95.06	94.76	95.78	89.75
90	92.57	93.09	92.81	91.54	95.53	95.39	95.96	91.26
100	92.61	94.28	93.27	92.02	95.67	95.27	96.12	91.44
Metrics								
Kappa Score	0.7021	0.6436	0.8463	0.6985	0.7912	0.6834	0.8969	0.7065
KL divergence	0.6321	0.5265	0.7242	0.5924	0.6934	0.5986	0.8621	0.6428
Precision	0.74	0.56	0.85	0.51	0.76	0.67	0.83	0.55
Sensitivity (Recall)	0.88	0.78	0.91	0.67	0.91	0.89	0.93	0.71
Specificity	0.61	0.69	0.72	0.64	0.64	0.72	0.79	0.56
F-Score	0.80	0.65	0.878	0.57	0.82	0.76	0.879	0.61
**ROC-AUC Score**	0.8476	0.9021	0.9275	0.8959	0.9021	0.9102	0.9840	0.9067

**Fig. 6:**
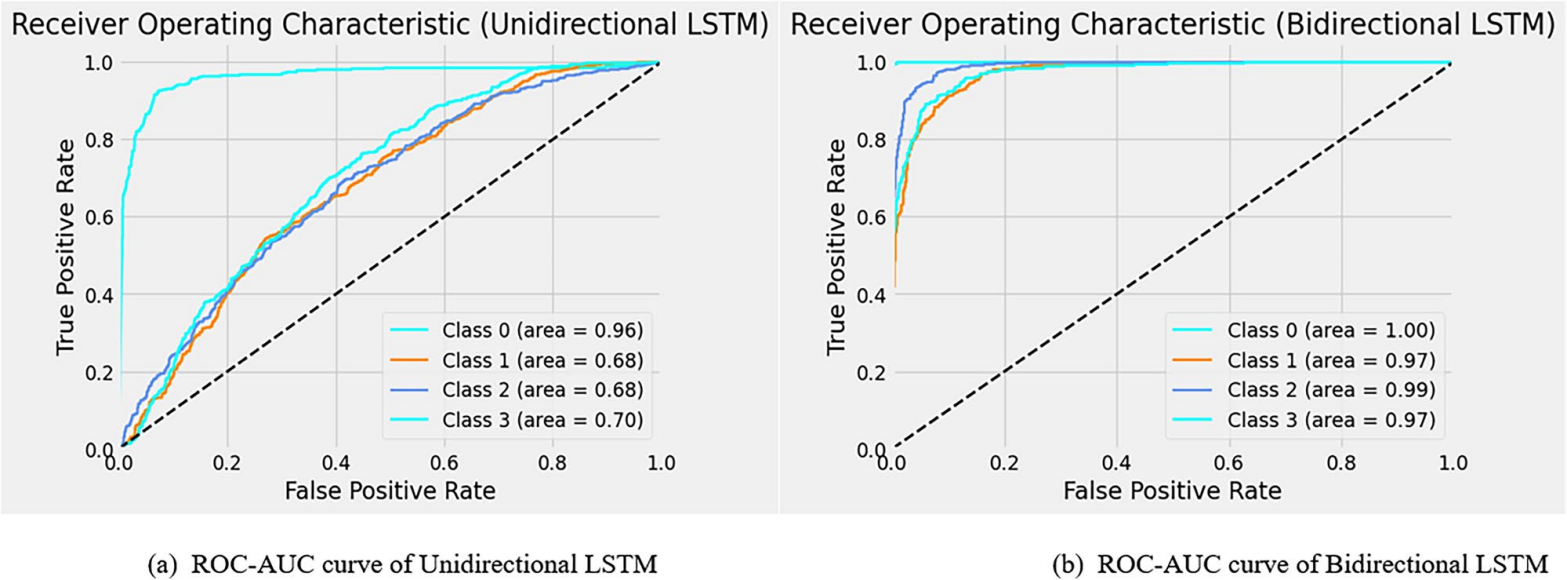
Results of ROC-AUC for RSFAS-Uni directional and RSFAS-Bidirectional LSTM.

Figure 6 shows the ROC curve for the proposed model compared with other models. The analysis of the ROC curves plot for the RSFAS-Unidirectional LSTM model as shown in Fig. 6a. **Class 0 (Stage-1):** AUC of 0.96—the model performs exceptionally well; **Class 1 (Stage-2) and Class 2(Stage-3):** AUC value: 0.68—poor to fair discriminative power; **Class 3 (Stage-4):** AUC value of 0.70—slightly better than random guessing. Similarly, Fig. 6b displays the ROC Curves for RSFAS-Bidirectional LSTM. **Class 0:** The AUC of 1.00 is indicative of exceptional performance; ** Class 1, Class 2, and Class 3:** AUC values of 0.97, 0.99, and 0.97, respectively shows healthy is added in Table 20.

On evaluating the performance of the proposed models, it was found that there is a variation in the calculated accuracies till the 40th epoch. After that, the accuracy and the value-loss are stabilized for batch sizes 10, 32, and 64. On considering the length of the paper the accuracy and value loss were given with four optimizers for Uni and Bidirectional LSTM for batch size 32 and batch size 64 in Tables 17 and 18 respectively. Even though the accuracy increases the computation time is recorded and found to be considerably higher for RSFAS-Bidirectional LSTM rather than the RSFAS-Unidirectional LSTM. The comparative execution time is depicted in Table 21.

**Table 21:** Execution time of classification and prediction accuracy for batch size-64.

Epochs	Parameters	RSFAS-Unidirectional		RSFAS-Bidirectional	
		Classification	Prediction	Classification	Prediction
5	Accuracy (%)	90.62	91.36	93.14	92.02
	Value-loss	0.0057	0.0171	0.0069	0.0172
	Execution time-(Secs)	30.44		32.52	
10	Accuracy (%)	90.64	91.69	93.56	92.89
	Value-loss	0.0114	0.0173	0.0139	0.0169
	Execution time-(Secs)	35.25		39.32	
20	Accuracy (%)	90.75	91.97	93.71	93.34
	Value-loss	0.0243	0.6162	0.0095	0.0154
	Execution time-(Secs)	44.64		43.59	
30	Accuracy (%)	0.96	92.08	93.99	94.42
	Value-loss	0.0164	0.0165	0.0087	0.0156
	Execution time-(Secs)	48.86		48.95	
40	Accuracy (%)	91.64	92.85	94.33	95.63
	Value-loss	0.012	0.0153	0.0073	0.0144
	Execution time-(Secs)	54.75		52.67	
50	Accuracy (%)	91.77	92.91	94.49	96.29
	Value-loss	0.0125	0.0146	0.0107	0.01354
	Execution time-(Secs)	57.78		62.63	
60	Accuracy (%)	91.98	93.24	94.93	96.32
	Value-loss	0.0256	0.0135	0.0129	0.0123
	Execution time-(Secs)	63.81		73.72	
70	Accuracy (%)	92.69	93.56	95.37	97.86
	Value-loss	0.0107	0.0126	0.0111	0.0121
	Execution time-(Secs)	63.94		79.67	
80	Accuracy (%)	92.73	94.12	95.78	97.93
	Value-loss	0.0114	0.0118	0.0093	0.0112
	Execution time-(Secs)	65.38		82.32	
90	Accuracy (%)	92.81	94.76	95.96	98.01
	Value-loss	0.019	0.0114	0.0036	0.0112
	Execution time-(Secs)	73.45		89.95	
100	Accuracy (%)	**93.27**	**95.98**	**96.12**	**98.05**
	Value-loss	**0.0191**	**0.0144**	**0.0022**	**0.0112**
	Execution time-(Secs)	76.47		92.52	

Significant values are in bold.

From, Table 21, it is seen clearly, as the number of epochs increases, the execution time increases. Thus, the proposed model has the limitation of execution time and cost as the number of epochs increases. From various research directions, it was found that the ensemble technique can achieve higher classification and recognition performance as it combines the weak (base learner) with the predictive models, rather than a single model. So that is considered as the future work of the proposed model.

**Fig. 7:**
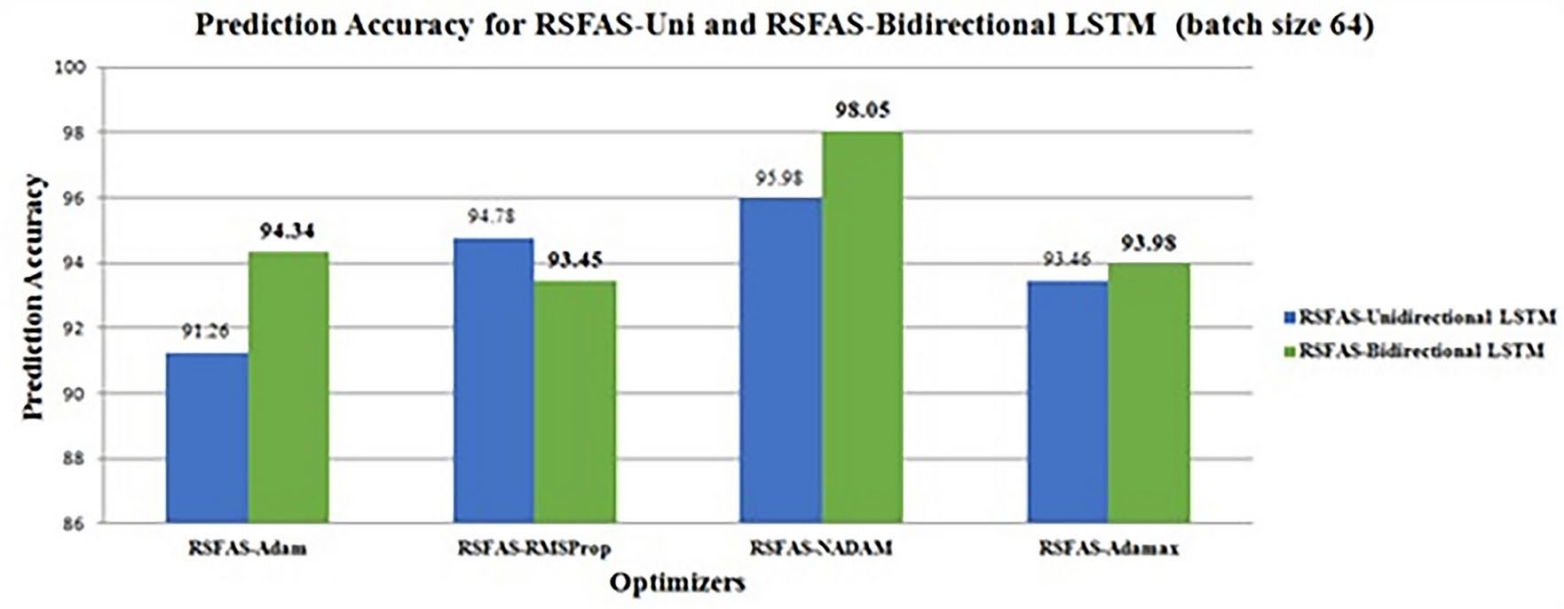
Prediction Accuracy of Unidirectional and Bidirectional LSTM model (Batch size-64).

Similarly, it is seen that the optimizer NADAM provides a better accuracy of 93.27% with feasible val-loss for RSFAS-Unidirectional LSTM. Similarly, the RSFAS-Bidirectional LSTM provides an accuracy of **96.12**% using NADAM optimizer with a validation loss of 0.0022. The trained model is now tested with the testing data, and the accuracies obtained for various optimizers are recorded and the prediction accuracy is shown in Fig. 7. Thus, it shows that the NADAM optimizer is best suited for the proposed work.

## Comparative analysis with the benchmarking models

**Fig. 8:**
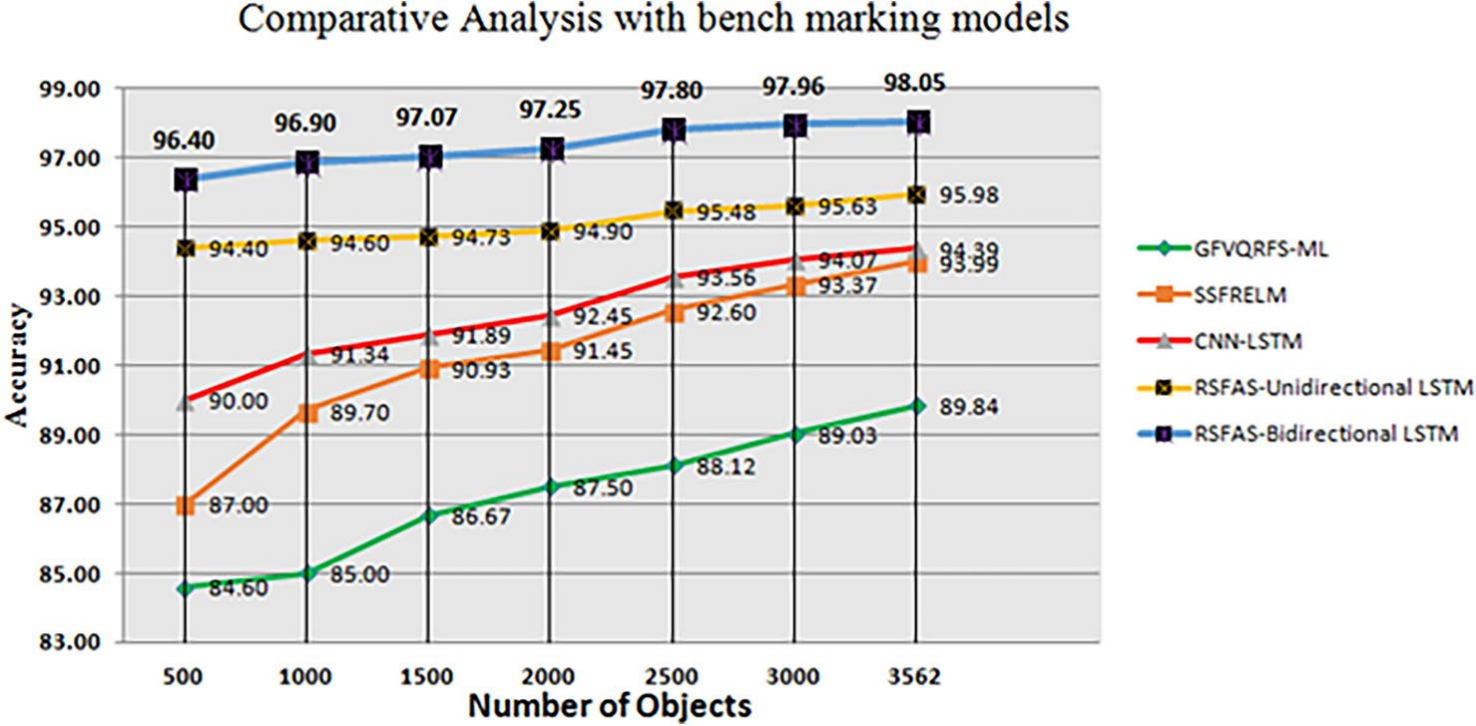
Comparative analysis with benchmarking models.

This section deals with the comparative analysis of the proposed models with the existing benchmarking techniques. Kumar and Halder^16^ proposed a hybrid model using greedy fuzzy vaguely quantified rough approach for feature (gene) selection (GFVQRFS) technique with machine learning for colorectal cancer prediction. Similarly, Kumar et al.^17^ proposed a semi-supervised fuzzy rough-based extreme learning machine (SSFRELM) method to enhance classification accuracy using fuzzy rough set theory to predict cancer. In addition, the proposed model was compared against deep learning techniques proposed by Tasdelen et al.^22^ on the CNN-LSTM model. The prediction accuracy against the number of objects with the models is tested using the testing data (3562 objects) and the obtained accuracies are provided in Table 22 and the same is depicted in Fig. 8. Table 21 shows that the proposed RSFAS-Uni directional model obtains 95.98% of prediction accuracy, whereas RSFAS-Bidirectional LSTM provides 98.05% accuracy. Thus the increase of 2.07% of accuracy provides a better prediction model than the compared models.

**Table 22:** Comparative analysis with benchmarking models.

No. of Objects	Kumar & Halder et al.^16^	Kumar et al.^17^	Tasdelen et al.^22^	Proposed models	
	GFVQRFS-ML	SSFRELM	CNN-LSTM	RSFAS-Unidirectional LSTM	RSFAS-Bidirectional LSTM
500	423 (84.60%)	435 (87%)	450 (90.00%)	472 (94.40%)	482 (96.40%)
1000	850 (89.70%)	897 (89.70%)	913 (91.34%)	946 (94.60%)	969 (96.90%)
1500	1300 (86.67%)	1364 (90.93%)	1378 (91.89%)	1421 (94.73%)	1456 (97.06%)
2000	1750 (87.50%)	1829 (91.45%)	1849 (92.45%)	1898 (94.90%)	1945 (97.25%)
2500	2203 (88.12%)	2315 (92.60%)	2339 (93.56%)	2387 (95.48%)	2445 (97.80%)
3000	2671 (89.03%)	2801 (93.37%)	2822 (94.07%)	2864 (95.63%)	2939 (97.96%)
3562	3200 (89.84%)	3348 (93.99%)	3362 (94.39%)	**3419 (95.98%)**	**3493 (98.05%)**

Significant values are in bold.

## Discussion on overall survival analysis of the patients at every stage

Survival analysis with the proposed prediction model has proven to be a better predictor of colorectal cancer based on the input data and utilization of the RSFAS. The analysis shows that the proposed model can make meaningful predictions and conclusions based on stages. The collected dataset shows the Duke stages as follows: Stage 1 (tumor invasion), Stage 2 (tumor occupation through the bowel wall without linking lymph nodes), Stage 3 (lymph node connection), and Stage 4 (Prevalent metastasis). The prediction analysis of the proposed model involves stage-based^32,33^prediction with significant features representing different gene expression levels in the collected dataset as shown in Table 23.

**Table 23:** Stage-based prediction with significant features and different gene expression levels.

Gene	Stage-1	Stage-2	Stage-3	Stage-4
1553153_at $(a_7)$	3.73–5.0	3.31–4.28	3.5–4.54	3.61–5.32
1552306_at $(a_8)$	3.38–5.08	3.23–7.26	3.63–5.73	2.76–4.72
1552318_at $(a_9)$	4.85 –4.19	3.47–4.73	3.4–4.87	3.69–4.58
1552390_a_at $(a_{10})$	3.05–4.77	3.2–4.62	2.79–4.38	3.02–4.34
1552486_s_at $(a_{11})$	5.52–7.55	5.51–6.35	5.34–6.72	6.21–7.59
1552633_at $(a_12)$	4.2–5.3	3.98–5.16	4.32–5.29	4.13–4.73
1552767_a_at $(a_{13})$	3.59–4.46, 6.73–8.67	3.69–4.84, 8.31–9.57	4.43, 7.13–10.35	8.05–9.02
1553177_at $(a_{14})$	2.99–4.24	5.29–7.58	5.01–7.18	5.78–8.31
1552807_a_at $(a_{15})$	5.87–7.92	2.98–5.09	2.86–4.23	2.98–3.81
1553220_at $(a_{16})$	4.16–5.55	3.72–5.78	3.59–5.26	4.16–5.69
1554001_at $(a_{17})$	3.7–5.48	3.8–6.23	3.48–5.69	3.99–5.72
1554004_a_at $(a_{18})$	3.96–6.26	4.4–5.95	3.93–5.74	4.26–5.48
1554007_at $(a_{19})$	3.23–8.03	3.33–5.92	4.03–7.61	3.21–7.59
1554095_at $(a_{20})$	5.15–7.04	5.07–7.01	4.46–7.81	5.29–6.95
1554102_a_at $(a_{21})$	4.05–5.85	3.45–4.82	5.33–6.02	4.09–6.55
1554341_a_at $(a_{22})$	4.24–5.31	5.03–5.10	3.51–5.07	3.67–5.75
1554485_s_at $(a_{23})$	4.38–6.47	4.35–8.52	3.99–6.75	4.54–7.48
Likelihood ratio	**328.34**	**344.3**	**276.07**	**307.6**

The analysis from Table 23, it is found that the genes $(a_7)$ to $(a_{23})$ contribute to the stage prediction process. As these genes are identified as significant attributes, the Weibull distribution helps predict the patients’ survival rate, Maximum Likelihood estimate (MLE), 95% Lower Confidence Interval (CI), and 95% Upper Confidence Interval (CI) of survival for 1 year to 10 years as represented in Tables 24 and 25. The gene mutation analysis provides the Weibull stage-based survival analysis for one year as follows:

- Stage 1 has the predicted objects of a total of 2856, with existing events of 90, and the observed events total 85, with a 1-year survival rate of 96
- Stage-2 has the predicted objects of a total of 2845, with existing events of 89, and the observed events are a total of 91, with a survival rate of 96
- Stage-3 has the predicted objects of a total of 2880, with existing events of 87, and the observed events are a total of 92, with a survival rate of 95
- Stage-4 has the predicted objects of a total of 2981, with existing events of 92, and the observed events are a total of 93, with a survival rate of 96

**Fig. 9:**
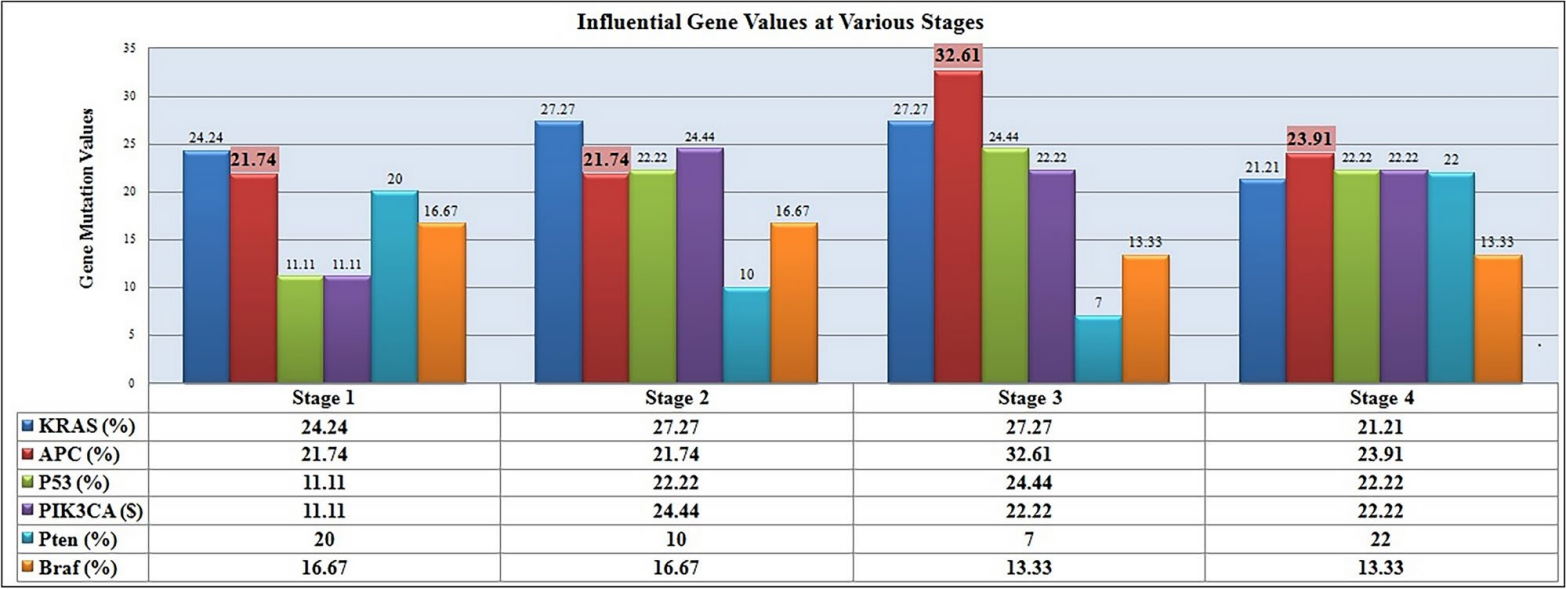
Influential gene values at various stages.

**Table 24:** Survival rate of patients with RSFAS-Weibull distribution.

Stages	Existing event(%)	Observed event(%)	Weibull-likelihood Ratio	C-index
Stage-1 (Objects:2856)	85	82	328.34	0.64
Stage-2 (Objects:2845)	91	87	344.30	0.60
Stage-3 (Objects:2880)	92	87	276.07	0.62
Stage-4 (Objects:2981)	93	89	307.60	0.61
Total Objects=11562				

**Table 25:** Survival analysis of RSFAS-Weibull distribution.

N=11,562 Objects	Survival analysis			MLE of Survival			95% Lower CI of Survival			95% Upper CI of Survival		
	1 Yr	5 Yrs	10 Yrs	1 yr	5 Yrs	10 Yrs	1 Yr	5 Yrs	10 Yrs	1 Yr	5 Yrs	10 Yrs
Stage-1	96%	80%	20%	99%	83%	99%	99%	81%	48%	99%	95%	57%
Stage-2	96%	67%	10%	97%	68%	37%	96%	67%	38%	97%	69%	39%
Stage-3	95%	63%	8%	96%	64%	35%	96%	63%	35%	98%	66%	36%
Stage-4	96%	62%	7%	95%	63%	35%	95%	67%	36%	96%	64%	36%

From Table 23, based on the frequency count of the gene that contributes to each stage is represented in Table 26, and the same is represented in Fig. 9. From Fig. 9, it is seen that the KRAS was identified between (24 to 28), the possibility of colorectal cancer to be of Stage 1 or Stage 2, whereas if the APC value is greater than 30, the possibility is Stage 3. If the gene value lies above 20 for PIK3CA, Pten, and P53 with APC greater than 20, then the patient may be of Stage 4. The domain experts recommended checking for the APC and KRAS gene values for the first level of treatment.

**Table 26:** Gene mutation with respect to stages in CRC.

Stages	KRAS (%)	APC (%)	P53 (%)	PIK3CA (%)	Pten (%)	Braf (%)
1	24.24	21.74	11.11	11.11	20.00	16.67
2	27.27	21.74	22.22	44.44	10.00	16.67
3	27.27	32.61	44.44	22.22	7.00	13.33
4	21.21	23.91	22.22	22.22	22.00	13.33

From the above analysis, when a person is reported with the symptoms of CRC, such as familial adenomatous polyposis (FAP) and with gastric adenocarcinoma, then it is advised to have the gene mutation test. As the APC takes a range of 22–33% of stages detection from 1 to 4, it is advised to check the APC gene mutation test. Also, the KRAS gene mutation falls within the range of 24–27% for the four stages of CRC, it is also recommended either for the biopsy of the colorectal adenocarcinoma tumor. Thus the following prediction table represents the range of the mutation recommendations for the various stages, which helps new patients reported with the CRC symptoms.

## Survival analysis with risk

The analysis of survival with risk using Weibull distribution has been carried out using the Maximum Likelihood Estimation (MLE) and with performance statistical inference such as 95% of Lower confidence and 95% of Upper confidence intervals to conclude the survival data. The above Table 25 gains insights into the underlying mechanisms of colorectal cancer disease and makes informed decisions about treatment and patient care.

**Fig. 10:**
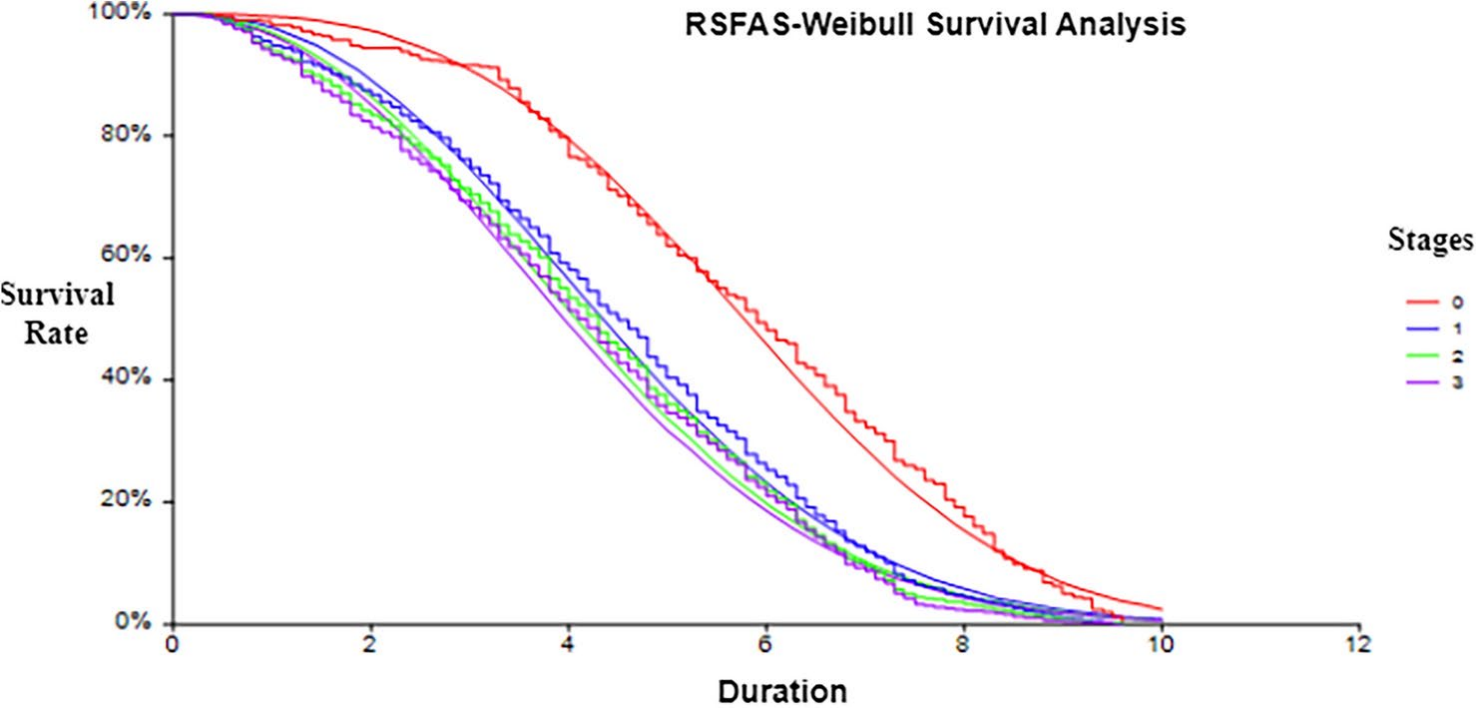
RSFAS-Weibull survival analysis for 10 years at various stages.

The analysis was calculated with the duration and the stages from Fig. 10. From the above Table 25, when the patient was diagnosed with Stage 1, the probability of survival, lies from 96 to 20% for 1–10 years, (ie) the risk rate is very low during the 1st year, whereas it is getting higher for 5 years and 10 years. Similarly, the probability of survival lies from 96 to 10% for Stage 2, (ie) the expected survival rate is 67% for 5 years and 10% of survival for 10 years. In addition, for Stage-3 and Stage-4, the survival is expected to be 62–7% which is almost similar in both cases. Also, the influential gene mutation is given to have more idea about the mutating genes for various stages.

**Clinical Contributions:**

- **Improved Survival Prediction:** By integrating clinical data (such as age, gender, tumor location, radiotherapy, and chemotherapy treatments) with gene-related data (including mutations in APC, KRAS, PTEN, P53, PIK3CA, and BRAF), our model significantly enhances the accuracy of patient survival predictions across different stages of CRC (Stages 1, 2, 3, and 4).
- **Personalized Treatment Insights:** The proposed study provides valuable insights into how specific gene mutations correlate with patient outcomes, enabling more personalized treatment plans and targeted therapies.    

**Technical Contributions:**

- **Comprehensive Feature Selection:** VIF is applied to the collected dataset helps us to find the significant attributes based on multicollinearity.
- **Handling Uncertainty:** Rough Set on Fuzzy Approximation Space helps in handling the uncertainty and to quantify the data to ensure consistency.
- **Stage-Specific Analysis:** Our model provides stage-specific analyses, offering detailed insights into how the influence of key genes varies across CRC on employing the dataset to Uni and Bidirectional LSTM. This granularity aids in understanding the disease progression and tailoring interventions accordingly.

These contributions helps in identifying the severity of the disease and to take immediate actions to save the affected patients on time.

## Conclusion with future enhancement

The paper aims to predict stage-based survival analysis of colorectal cancer patients using gene expression data. Since the real-time data suffers from uncertainty, it is handled by using a rough set on fuzzy approximation space. In addition, the survival analysis process was taken care of using Weibull distribution. The feature selection process was taken care of using the Variance Inflation Factor (VIF). Upon pre-processed data, the consistent data undergoes training and testing using various optimizers with Unidirectional and Bidirectional LSTM. The optimization of the proposed work was taken care by employing four optimizers such as Adam, NADAM, RMSProp, and Adamax. The NADAM optimizer was found to yield better-predicted accuracy with minimal loss and thus reduce the overfitting issues. The hyperparameters utilized for the proposed Unidirectional and Bidirectional models were compared with existing benchmarking models, showing that the proposed work predicts better with the prediction accuracy of **98.05%**. The proposed model is effective for forecasting the survival rate using the gene expression data of stage based colorectal cancer. However, the proposed model has the limitation of training and testing of a high number of epochs with considerable computation time. As the number of objects increases, the accuracy increases but at the same time, the computation time also increases. Thus, the proposed model can be enhanced using the ensemble technique to mitigate the issue of computation time. The ensemble technique trains the dataset concurrently by identifying the base learner classifier followed by the subset selection method using the stacking technique for classification and prediction, Thus, this process has been planned for future work possibly the stacked LSTM will be utilized for the classification and prediction process.

## Data Availability

The dataset was collected from Organization of Queen’s University Belfast from Centre for Cancer Research and Cell Biology and available open source by National Centre for Biotechnology Information (NCBI) for the colorectal cancer patients available open source since Dec 2017. The dataset was characterized as clinical data and gene data using geo accession details Data will be made available from the corresponding author.
